# Hierarchical affinity landscape navigation through learning a shared pocket-ligand space

**DOI:** 10.1016/j.patter.2025.101371

**Published:** 2025-09-17

**Authors:** Bin Feng, Zijing Liu, Hao Li, Mingjun Yang, Junjie Zou, He Cao, Yu Li, Lei Zhang, Sheng Wang

**Affiliations:** 1 International Digital Economy Academy (IDEA), Shenzhen, China; 2 XtalPi Co., Ltd., Shenzhen, China; 3 Paul G. Allen School of Computer Science and Engineering, University of Washington, Seattle, WA, USA

**Keywords:** virtual screening, hit-to-lead optimization, affinity prediction, shared space, scaffold discrimination, pharmacophore ranking

## Abstract

The structure of the protein binding pocket governs the ligand binding affinity by providing crucial intermolecular interactions and spatial compatibility. While existing methods have leveraged these structural insights to advance affinity prediction, they often treat virtual screening and hit-to-lead optimization separately, mainly due to incompatible speed-accuracy requirements. However, these two tasks complement each other, and their integration enables broader chemical exploration while preserving focus on affinity-determining substructures. Here, we present ligand unified affinity (LigUnity), a foundation model for affinity prediction that jointly embeds ligands and pockets into a shared space. In particular, LigUnity learns coarse-grained active/inactive distinction through scaffold discrimination and fine-grained pocket-specific ligand preference through pharmacophore ranking. We demonstrate the effectiveness and versatility of LigUnity on eight benchmarks across six settings. In virtual screening, LigUnity outperforms 24 methods with >50% improvement and demonstrates robust generalization to novel targets. In hit-to-lead optimization, it achieves state-of-the-art performance across split-by-time, split-by-scaffold, and split-by-unit settings, emerging as a cost-efficient alternative to free energy perturbation. We further showcase how LigUnity can be employed in an active learning framework for tyrosine kinase 2 (TYK2) to efficiently find optimal ligands. Collectively, these results establish LigUnity as a versatile foundation model for affinity prediction, offering broad applicability across the drug discovery pipeline.

## Highlights

• LigUnity is a unified foundation model for virtual screening and hit-to-lead optimization
• Outperforms 24 methods with >50% improvement in virtual screening
• Approaches FEP+ accuracy at far lower cost in hit-to-lead optimization
• Achieves 10^6^ speedup compared to traditional docking methods like Glide-SP

## The bigger picture

Despite thousands of proteins in the human genome, only about 10% are currently bound to small molecules with high potency, highlighting the urgent need to discover active molecules for new therapeutic targets. As experimental affinity data accumulate rapidly, unprecedented resources are available for machine learning methods. However, most existing methods developed for identifying active molecules treat the valuable data in a coarse, binary fashion, simply classifying compounds as active or inactive for a given target. These approaches overlook the important information contained in these data—not just whether a molecule works but also how well it works and what subtle chemical differences make one compound more effective than another.

LigUnity is a foundation model designed for addressing this challenge. It learns a shared “language” of protein pockets and small molecules that captures both whether a molecule binds and how efficiently it does so, with improved efficiency and accuracy. LigUnity makes efficient exploration of vast chemical spaces possible and accelerates the search for new medicines.

## Teaser

LigUnity is a protein-ligand affinity foundation model, enabling both rapid virtual screening and precise hit-to-lead optimization. LigUnity learns a shared “language” for protein pockets and ligands, outperforming docking in screening and approaching FEP+ accuracy at far lower cost. It generalizes to new targets and new chemical scaffolds and achieves 10^6^ times the speedup compared to Glide-SP, offering a practical foundation for early-stage drug discovery.

## Introduction

The structure of the protein binding pocket, a spatially defined ligand binding cavity in proteins, serves as the foundation for computational drug design by determining non-covalent interactions and stereochemical complementarity with ligands.^1^ This structure enables accurate prediction of protein-ligand binding affinity, a key determinant of drug efficacy and target selectivity. Accurately predicting this critical binding affinity plays a key role in two sequential steps in target-based drug discovery: high-throughput virtual screening, which aims to discover active compounds that can bind to an interested human protein from large-scale chemical libraries,^2^^,^^3^^,^^4^ and hit-to-lead optimization, which aims to refine the structure of these active ligands to improve their binding affinity and drug-like properties.^5^^,^^6^ For virtual screening, docking methods have been widely used with several successful applications.^7^ Still, they suffer from the trade-off between the chemical search space size and computational cost.^8^ As for hit-to-lead optimization, physics-based calculation methods, including free energy perturbation (FEP)^9^ and molecular mechanics-generalized born surface area (MM/GBSA),^10^ have shown encouraging performance but are either poorly correlated with experimental binding affinities or require large computational resources for extensive sampling.^11^^,^^12^ To address the challenges faced by computational methods, in recent years, machine learning (ML) methods have been developed for both virtual screening (e.g., DrugCLIP^13^) and hit-to-lead optimization (e.g., ActFound^14^ and PBCNet^15^). These ML approaches achieve comparable performance to computational methods while offering significant improvements in computational efficiency, thereby making them highly efficient alternatives for large-scale applications.

Despite the encouraging performance of existing ML methods, virtual screening and hit-to-lead optimization are often studied separately. However, these two tasks are interdependent and complementary to each other. A model solely focused on hit-to-lead optimization might be constrained within a limited chemical space, hindering its ability to generalize to ligands with novel chemical scaffolds. Conversely, a model developed exclusively for virtual screening might overlook crucial substructures that determine protein-ligand interactions, thus failing to distinguish between ligands with subtle structural differences. Several recent works (e.g., GenScore,^16^ PIGNet2,^17^ IGModel,^18^ and EquiScore^19^) have begun bridging the gap between these two tasks by data augmentation techniques like re-docking, cross-docking, and decoy generation. However, these approaches still face challenges in collecting structurally similar active ligand pairs, which is crucial for learning subtle substructural variations that affect binding affinity. Based on these observations, we hypothesize that a unified foundation model jointly addressing virtual screening and hit-to-lead optimization can leverage the synergy between these two tasks to enhance overall performance.

In this work, we propose LigUnity (ligand unified affinity), a protein-ligand affinity foundation model. The core innovation of LigUnity lies in its integrated capabilities for both broad screening and precise affinity prediction, achieved through a combination of scaffold discrimination and pharmacophore ranking. We jointly embed ligands and protein pockets into a shared space that captures their structural and chemical complementarity. For scaffold discrimination, LigUnity learns to differentiate between active and inactive ligands, providing insights into diverse protein-ligand interactions that help establish global structure-activity relationships across chemical scaffolds. For pharmacophore ranking, LigUnity refines the shared space by learning to order active ligands for each pocket, revealing how subtle structural differences affect binding affinity. With these computed embeddings, LigUnity enables rapid screening of large virtual libraries and efficient identification of active ligands, making it suitable for both virtual screening and hit-to-lead optimization tasks.

However, collecting a structure-aware dataset suitable for both tasks poses a significant challenge. While large-scale affinity datasets like BindingDB^20^ and ChEMBL^21^ provide abundant binding data, they lack the structural details necessary for identifying binding pocket structures. To address this, we introduce PocketAffDB, a comprehensive structure-aware binding assay database containing 0.8 million affinity data points across 0.5 million unique ligands and 53,406 pockets. PocketAffDB is organized by assays, in which all affinity measurements share the same experimental methods and are directly comparable. Building on this, we propose a simple but effective assay-guided pocket-matching method to assign a binding pocket structure to each protein-ligand pair, based on the observation that most assays are designed for a specific binding site of interest. To our best knowledge, PocketAffDB represents the largest affinity dataset that integrates bioassay data with binding pocket structure, providing a valuable resource for affinity prediction.

To validate the effectiveness of LigUnity, we conducted comprehensive evaluations on eight benchmarks across six settings. On three virtual screening benchmarks (DUD-E,^22^ Dekois,^23^ and LIT-PCBA^24^), LigUnity outperforms all 24 competing methods. When applied to unseen proteins, our model achieves consistent improvement, demonstrating its robust generalization ability to novel targets. For hit-to-lead optimization, LigUnity shows superior performance on two FEP benchmarks in both zero-shot and few-shot settings, suggesting that LigUnity can be potentially used as an efficient alternative to the costly FEP calculations.^9^ We further assessed LigUnity’s adaptability in split-by-time, split-by-scaffold, and split-by-unit settings on ChEMBL^21^ and BindingDB^20^ datasets, with LigUnity again outperforming eight competing methods. Moreover, in an active learning framework simulating multi-iteration drug discovery optimization, LigUnity successfully identifies ligands with optimal binding affinity in just a few iterations, illustrating its practical value to minimize the experimental cost in molecular optimization. Collectively, LigUnity serves as a foundation model for both virtual screening and hit-to-lead optimization, offering broad applicability across the drug discovery pipeline.

## Results

### Overview of LigUnity

**Figure 1:**
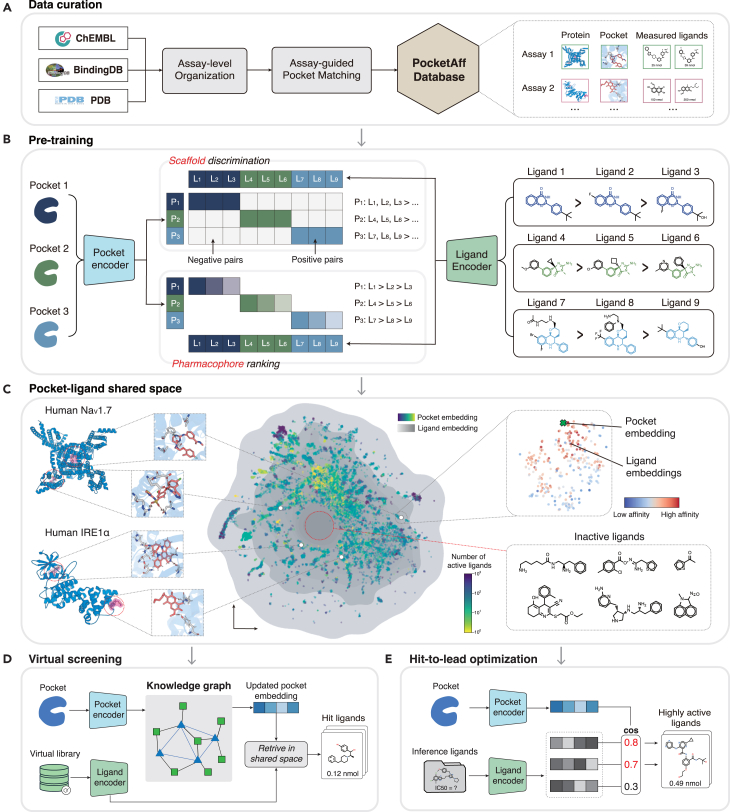
Overview of LigUnity (A) Data curation pipeline of PocketAffDB. (B) LigUnity first pre-trains the pocket and ligand encoders by a hierarchical affinity landscape navigation. It first projects pockets and ligands into a shared embedding space and then exploits scaffold discrimination to capture coarse-grained active/inactive distinction and pharmacophore rankings to refine the shared embedding space by aligning it with subtle affinity differences. (C) Uniform manifold approximation and projection (UMAP) plot showing ligand and pocket embeddings derived by LigUnity. (D) For virtual screening, LigUnity first refines the query pocket embedding by a graph neural network, and the updated pocket embedding is then utilized to retrieve hit ligands in the pocket-ligand shared space. (E) For lead optimization, LigUnity is directly used to rank unmeasured ligands based on cosine similarity between pocket and ligand embeddings and find highly active ligands among them.

LigUnity is a protein-ligand affinity foundation model for both virtual screening and hit-to-lead optimization, employing 3D binding pocket structure to predict protein-ligand binding affinities. To train the model, we developed PocketAffDB, a comprehensive structure-aware dataset curated from large-scale experimental affinity databases (BindingDB^20^ and ChEMBL^21^) and PDB (Figure 1A). We first collect affinity data into assays and then assign a binding pocket structure to each protein-ligand pair using assay-guided pocket matching (see methods). To our knowledge, PocketAffDB represents the largest integrated affinity dataset combining bioassay data with structural pocket information, comprising 0.8 million affinity data points spanning 0.5 million unique ligands, 53,406 pockets, and 26,748 assays.

In the pre-training stage, LigUnity learns a pocket-ligand shared space to capture structural and chemical complementarity between pockets and ligands. We implement this through an integrated framework combining scaffold discrimination and pharmacophore ranking (Figure 1B). The scaffold discrimination module learns to distinguish active/inactive compounds by focusing on structural differences in the chemical scaffold, creating an embedding space where positive pocket-ligand pairs are attracted while dissimilar negative pairs are repelled. The pharmacophore-ranking component then refines this embedding space through fine-grained alignment with subtle affinity differences by focusing on the pharmacophore of ligands, where similarity rankings between ligand and pocket embeddings are used to predict relative binding affinities. These two modules complement each other and thus are employed simultaneously in the pre-training stage.

After pre-training, the embedding space of LigUnity demonstrates meaningful hierarchical patterns (Figure 1C). At the coarse-grained level, ligands binding to the same pocket form visible clusters, whereas ligands targeting different pockets of the same protein are well separated, indicating that embeddings of LigUnity can capture coarse-grained binding site information, showing potential for virtual screening. At the fine-grained ligand level, the embedding space shows that ligands with higher binding affinity values are closer to their target pockets, indicating that LigUnity learns pharmacophore-level differences affecting binding affinity, demonstrating the ability for hit-to-lead optimization.

During inference, LigUnity is adapted separately for virtual screening and hit-to-lead optimization to leverage task-specific data. For virtual screening, LigUnity employs a graph-based approach to utilize large-scale protein-ligand interactions in existing databases (Figure 1D). Specifically, we first built a large-scale knowledge pocket-ligand graph, containing 0.83 million pocket-ligand edges with known interactions and 16 million pocket-pocket edges based on structural similarity. We then applied a graph neural network to refine the query pocket embedding by aggregating neighboring information. For hit-to-lead optimization, LigUnity directly gives predictions for unmeasured ligands by simply computing cosine similarity between pocket and ligand embeddings (Figure 1E). We can also enhance prediction accuracy by fine-tuning on a few experimentally measured ligands, which are often available for hit-to-lead optimization. Through modeling the affinity landscape in an embedding space, LigUnity substantially reduces the computational cost during inference, achieving six orders of magnitude speedup compared to traditional docking methods.

### LigUnity improves virtual screening

**Figure 2:**
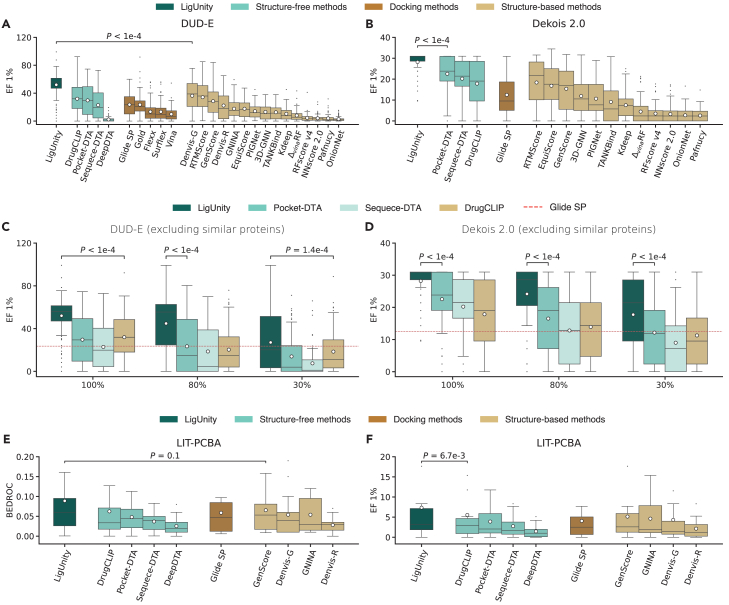
Evaluation on virtual screening (A and B) Boxplots comparing LigUnity and competing methods in the DUD-E and Dekois-2.0 benchmarks in terms of enrichment factor (EF) 1%. The mean values (white dots) are calculated across *n* = 102 and 81 targets for the DUD-E and Dekois-2.0 benchmarks, respectively. (C and D) Boxplots comparing LigUnity and structure-free methods in the DUD-E and Dekois-2.0 benchmarks in terms of EF 1% using different protein training sets. The *x* axis denotes the maximum sequence similarity between the training and test sets. The mean values (white dots) are calculated across *n* = 102 and 81 targets for the DUD-E and Dekois-2.0 benchmarks, respectively. (E and F) Boxplots comparing LigUnity and competing methods on the LIT-PCBA benchmark in terms of EF 1% and boltzmann-enhanced discrimination of receiver operating characteristic (BEDROC) score (α = 80.5). The mean values (white dots) are calculated across *n* = 15 targets. (A–F) *p* values indicate the significance for LigUnity outperforming the best comparison approach in a one-sided Wilcoxon test.

We first evaluated LigUnity for virtual screening on 102 protein targets from the DUD-E benchmark and 81 protein targets from the DEKOIS 2.0 benchmark.^22^^,^^23^ These two benchmarks cover a diverse set of targets spanning enzymes, ion channels, G-protein-coupled receptors (GPCRs), and transcription factors (Figures S1 and S2). We compared LigUnity with 24 different competing methods, including molecular docking methods,^25^^,^^26^ structure-based methods,^16^^,^^27^^,^^28^ and structure-free methods,^13^^,^^29^ and observed that LigUnity outperformed all competing methods in both benchmarks (Figures 2A, 2B, and S3–S6; Table S1). Specifically, when compared to the best competing structure-based methods, Denvis-G^28^ and RTMScore,^27^ LigUnity still achieves over 50% improvements in the enrichment factor (EF) 1% (*p* < 10^−9^). Meanwhile, once the embeddings are computed, LigUnity is 10^6^ times faster than Glide-SP in the screening speed, as LigUnity does not need docking poses (Figure S7). To assess the ability of LigUnity to generalize to unseen proteins, we excluded training proteins that are similar to any test protein in terms of sequence similarity (Figures 2C, 2D, S8, and S9). When using a stringent threshold of 30% sequence similarity, LigUnity still significantly outperforms both DrugCLIP and the commercial docking software Glide-SP (*p* < 0.05), demonstrating its generalizability to novel proteins.

Next, we evaluated LigUnity on the LIT-PCBA^24^ benchmark, which contains experimentally measured affinities for active and inactive ligands across 15 targets. Compared to DUD-E and DEKOIS, LIT-PCBA presents a more challenging setting for virtual screening, as it contains more inactive ligands that are structurally similar to active ligands. In contrast, DUD-E and DEKOIS utilize experimentally unverified decoys from the public compound database (ZINC)^30^ while excluding decoys structurally similar to active ligands to avoid potential false negatives, which may make them relatively easy for machine learning models. Furthermore, LIT-PCBA maintains a 1:1,000 active-to-inactive ratio, which better reflects the practical conditions encountered in real-world drug discovery. Nevertheless, LigUnity still achieves superior performance on the more challenging LIT-PCBA benchmark (Figures 2E and 2F). The improvement is also achieved when excluding training proteins that have large sequence similarity to any test protein using varying thresholds (Figure S10), reassuring the effectiveness and generalizability of LigUnity for virtual screening.

Finally, we conducted ablation studies to study the contribution of each module in LigUnity (Figures S11–S13). First, we found that ablating the scaffold discrimination component leads to a performance drop of over 60% in terms of EF 1%, confirming its crucial role in distinguishing active ligands from decoys in virtual screening tasks. Second, the ablation of the heterogeneous graph neural network (H-GNN) leads to consistent decreases in performance, demonstrating its capability to utilize information from the pocket-ligand heterogeneous graph to improve the predictive power. Furthermore, we found that the pharmacophore ranking is particularly effective on LIT-PCBA among three benchmarks (Figure S13), as it helps LigUnity distinguish subtle structural differences among similar ligands that are crucial to their affinity scores. Together, our ablation studies demonstrate the importance of all three key technical ideas proposed by LigUnity, necessitating the joint optimization of virtual screening and hit-to-lead optimization.

### LigUnity improves hit-to-lead optimization

Hit-to-lead optimization aims to optimize the structure of the ligand for improved affinity by ranking candidate ligands that are commonly structurally similar to initial hits identified to be active. After observing the substantial improvement of LigUnity in virtual screening, especially its ability to capture ligand structure differences, we next investigated whether LigUnity can be applied to hit-to-lead optimization. We evaluated LigUnity on two FEP benchmarks (JACS^9^ and Merck^31^) to predict binding free energies, which are the most widely used indicators for hit-to-lead optimization. These two benchmarks contain 16 targets, and each target has an average of 29 experimentally measured ligands. Following previous work,^14^ assays similar to those included in the FEP benchmarks are excluded in the pre-training stage to avoid potential data leakage (see methods). Ligands in the FEP benchmarks are also removed from the pre-training data.

**Figure 3:**
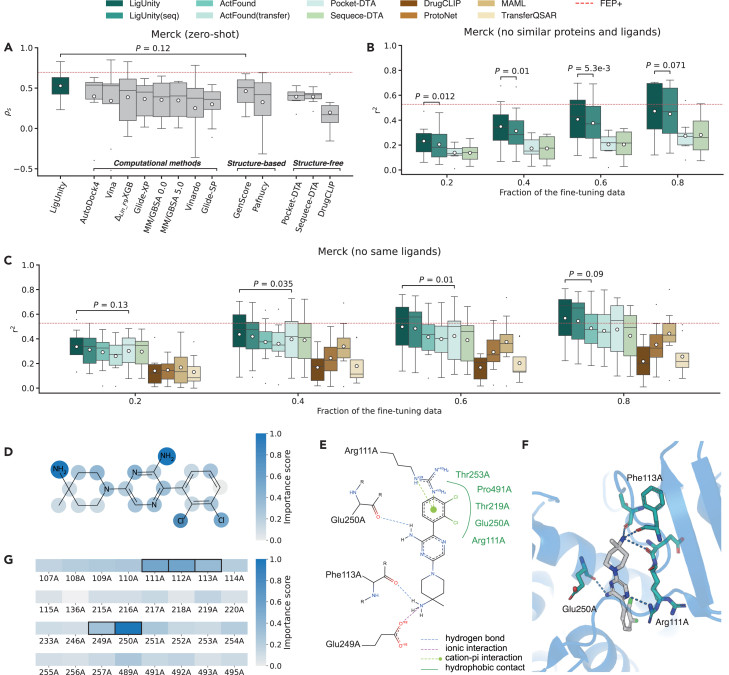
Evaluation on hit-to-lead optimization (A) Boxplot comparing the binding affinity prediction on the Merck benchmark in terms of Spearman’s rank correlation (*ρ*_*s*_) in the zero-shot setting. The mean values (white dots) are calculated across *n* = 8 targets. (B and C) Boxplots comparing the binding affinity prediction across different settings on the Merck benchmark in terms of *r*^2^ when 20%, 40%, 60%, and 80% of the experimental binding affinities are used for fine-tuning. The mean values (white dots) are calculated across *n* = 8 targets. (D) Case study on the SHP2 target showing the importance score of each ligand atom predicted by LigUnity. (E and F) 2D (E) and 3D (F) interaction graph showing the non-covalent interaction between the ligand and pocket (PDB: 5EHR). (G) Case study on the SHP2 target showing the importance score of each pocket residue predicted by LigUnity. The top five predicted key residues are highlighted with black boxes. (A–C) *p* values indicate the significance for LigUnity outperforming the best comparison approach in a one-sided Wilcoxon test.

In the zero-shot setting, where the model does not use any measured ligands for test proteins during pre-training and fine-tuning, our model surpasses existing computational methods (e.g., Glide-SP^25^ and MM/GBSA^32^), structure-based methods (e.g., GenScore^16^), and structure-free methods (e.g., DrugCLIP^13^) (Figure 3A) on 8 targets of the Merck FEP benchmark, demonstrating the effectiveness of our method. To further examine the performance of LigUnity in hit-to-lead optimization, we studied three more challenging settings: (1) a no similar ligands setting, where training ligands with more than 50% Tanimoto similarity to any test ligand are excluded; (2) a no similar proteins setting, where training proteins with more than 30% sequence similarity to any test protein are excluded^33^; and (3) a no similar ligands and proteins setting, where the criteria of the first two settings are applied to exclude similar proteins or ligands. LigUnity again consistently outperforms competing methods across all three settings (Figure S14).

In real-world drug discovery, binding data for a few ligands of interest are often experimentally measured. A successful hit-to-lead optimization model should be able to leverage these binding data to enhance its predictions. To examine the ability to employ these data, we fine-tuned LigUnity and competing methods with varying proportions of ligand binding data. We observed that LigUnity consistently outperforms competing methods across all four settings (Figures 3B, 3C, and S15; Table S2). Even under the most challenging setting where both similar ligands and proteins are excluded, LigUnity achieves an *r*^2^ of 0.472 on the Merck benchmark after fine-tuning on 80% of ligands (23.2 ligands on average for each target), reaching performance comparable to FEP+(OPLS4) (*r*^2^ = 0.528), a leading commercial computational software for calculating the binding free energy. These results demonstrate the practical value of LigUnity for affinity prediction, offering an accurate and efficient alternative to resource-intensive computational chemistry approaches.

Similar to our analyses in the virtual screening, we conducted ablation studies to investigate the contribution of each component in LigUnity (Figure S16). Our results demonstrate that excluding the scaffold discrimination or the pharmacophore-ranking component leads to substantial performance drops. Notably, ablating the pharmacophore ranking leads to a performance drop of over 50% in both zero-shot and few-shot settings, highlighting the effectiveness of the ranking objective in capturing the relative activity differences for hit-to-lead optimization tasks. To verify that LigUnity captures meaningful interaction patterns, we systematically tested it on FEP benchmarks by masking interaction-critical residues. Key residues were identified from PDB structures using ProteinPlus.^34^ The results show that masking strong H-bond/ionic bond residues caused significant performance drops $\left(\Delta \rho_{s}=-6.34\%\right)$ vs. minimal impact from random masking $\left(\Delta \rho_{s}=-0.46\%\right)$ (Table S3), demonstrating LigUnity’s ability to rely on protein-ligand interactions to achieve good performance.

Finally, to help understand the structural significance of individual atoms and residues involved in the interaction, we calculated the importance score of each atom and protein pocket residue on the SHP2 target by masking them individually (Figures 3D–3G and S17; see methods). By examining the atom importance scores, LigUnity correctly identifies two amino groups (-NH2) as key contributing factors to the predicted binding affinity (Figure 3D), aligning with the crystallographic observations of their hydrogen bonding and ionic interactions (Figures 3E and 3F). Furthermore, using protein residue importance scores, the model correctly highlights the crucial role of Glu250E in hydrogen bonding, along with the polar interactions involving Phe113A, Arg249A, and Arg111A (Figure 3G). Although the pose-free nature of our approach results in certain limitations, such as underestimating the contributions from specific hydrophobic cavity residues (Thr253A and Pro491A), LigUnity successfully identifies key interaction patterns. These results suggest that LigUnity can serve as an interpretable computational tool for studying structure-activity relationships and guiding hit-to-lead optimization.

### LigUnity as a versatile foundation model for different applications

To further explore the ability of LigUnity as a foundation model for affinity prediction, we next sought to evaluate its performance on assays from ChEMBL^21^ and BindingDB,^20^ two comprehensive public databases containing experimentally measured binding affinities covering various aspects of assay types, such as protein-based, cell-based, cell-membrane-based, and subcellular-based assays.^20^^,^^21^ We first used a split-by-time setting to evaluate the performance, where the model is pre-trained using 18,552 assays released before March 2019 and tested on 161 assays released after March 2019 in ChEMBL and BindingDB. Each test assay has 48.6 ligands on average. To avoid potential data leakage, we further excluded test assays whose targets exist in the pre-training data.

**Figure 4:**
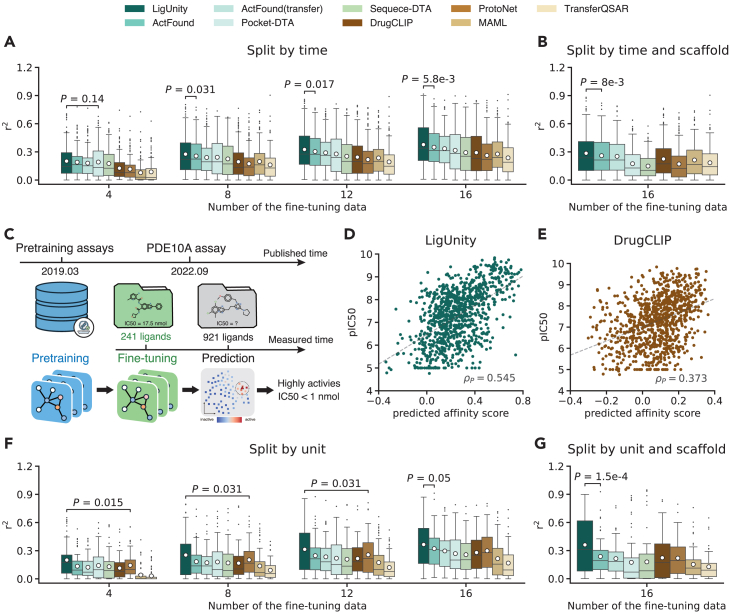
Evaluation on diverse settings (A and B) Boxplots comparing the binding affinity prediction on the split-by-time setting in terms of *r*^2^ when 4, 8, 12, and 16 experimentally measured ligands are used for fine-tuning. The mean values (white dots) are calculated across *n* = 161 assays. (C) Experimental setting for case study on the phosphodiesterase 10A (PDE10A) target. (D and E) Scatterplots comparing the predicted affinity score and pIC50 = −log_10_IC50 for LigUnity (F) and DrugCLIP (G) when fine-tuned with the first measured 20% ligands. (F and G) Boxplots comparing the binding affinity prediction on the split-by-unit setting in terms of *r*^2^ when 4, 8, 12, and 16 experimentally measured ligands are used for fine-tuning. The mean values (white dots) are calculated across *n* = 65 assays. (A, B, D, E, F, and G) *p* values indicate the significance for LigUnity outperforming the best comparison approach in a one-sided Wilcoxon test.

LigUnity attains consistent improvement using varying numbers of fine-tuning data ranging from 4 to 16 (Figures 4A and S18). When compared to ActFound,^14^ the best-competing method specifically designed for few-shot affinity prediction, LigUnity achieved an average improvement of 8.5% in terms of *r*^2^. Since ActFound is a ligand-based model without considering pocket information, our substantial improvement reflects the effectiveness of using protein pocket information for affinity prediction. In a more challenging split-by-scaffold setting where test ligands have distinct scaffolds from training ligands, LigUnity again obtains the best performance (Figure 4B), demonstrating its strong generalizability to unseen chemical scaffolds.

Next, we conducted a case study on phosphodiesterase 10A (PDE10A), a verified therapeutic target for antipsychotic therapy with no approved drugs, to assess the applicability of LigUnity in the real-world drug discovery pipeline. This dataset was released by Roche in 2022, comprising 1,162 ligands with the experimentally measured binding affinity and timestamp of each experiment.^35^ We used a split-by-time setting for ligands in the PDE10A dataset, where the first measured 20% ligands are used for fine-tuning and the remaining 80% are used for testing (Figure 4C). To ensure a rigorous split-by-time evaluation, we also restricted the pre-training data to the assays released before March 2019. To this end, our fine-tuned model achieves a Pearson correlation of 0.55 on the test set (Figure 4D), substantially outperforming the 0.37 Pearson correlation of DrugCLIP (Figure 4E). Building on this performance, our model demonstrates a strong ability to identify highly potent ligands, successfully identifying 4 highly active ligands (IC50 < 1 nmol) among the top-10 predicted ligands (Figure S19) and 24 highly active ligands among the top-50 predicted ligands (compared to 11 for DrugCLIP). Collectively, these results demonstrate the superior capability of LigUnity in identifying highly potent ligands, holding the potential to reduce the cost of hit-to-lead optimization.

Finally, we studied a split-by-unit setting where the test assays use percentage (%) units, a widely used format (24.4% in ChEMBL) in practical drug design with limited exploration in ML methods. Assays with percentage units pose challenges for affinity prediction, as they distribute differently from pre-training assays with molar concentrations (e.g., nmol) or density units (e.g., μg/mL). We hypothesize that our pharmacophore-ranking approach enables LigUnity to be robust against assays with different units, as it aims to learn the pocket-specific ligand ranking instead of the absolute affinity value. To this end, we examined a split-by-unit setting by using ChEMBL assays reporting activities in percentage (%) as test assays and assays with defined molar concentration or density units as pre-training assays. This test set contains 65 assays, and each assay has, on average, 30.5 ligands. When fine-tuned with different numbers of ligands (4–16), LigUnity consistently surpasses all competing methods (Figures 4F, 4G, and S18). Notably, compared to the regression model Pocket-DTA, LigUnity shows a 40.2% improvement in this split-by-unit setting, substantially higher than the 13.8% improvement in the split-by-time setting, demonstrating the generalization ability of the pharmacophore-ranking approach across different measurement units, reassuring LigUnity as an effective foundation model for drug discovery.

### LigUnity boosts the active learning framework for drug discovery

In the real-world drug discovery pipeline, multiple iterations of molecular optimization are often required due to limited experimental resources and the difficulty in accurately predicting protein-ligand binding affinity. As a result, active learning is often exploited in this process. Building on this, we explored how LigUnity can be integrated with active learning to optimize ligands for tyrosine kinase 2 (TYK2), a therapeutic target for autoimmune diseases. We employed a dataset that contains binding free energy for 10,000 ligands calculated using FEP, consuming approximately 80,000 GPU hours (9.1 GPU years).^36^ Following established active learning protocols,^37^ we aimed to identify highly active ligands with limited computational resources in terms of the amount of FEP calculations.

**Figure 5:**
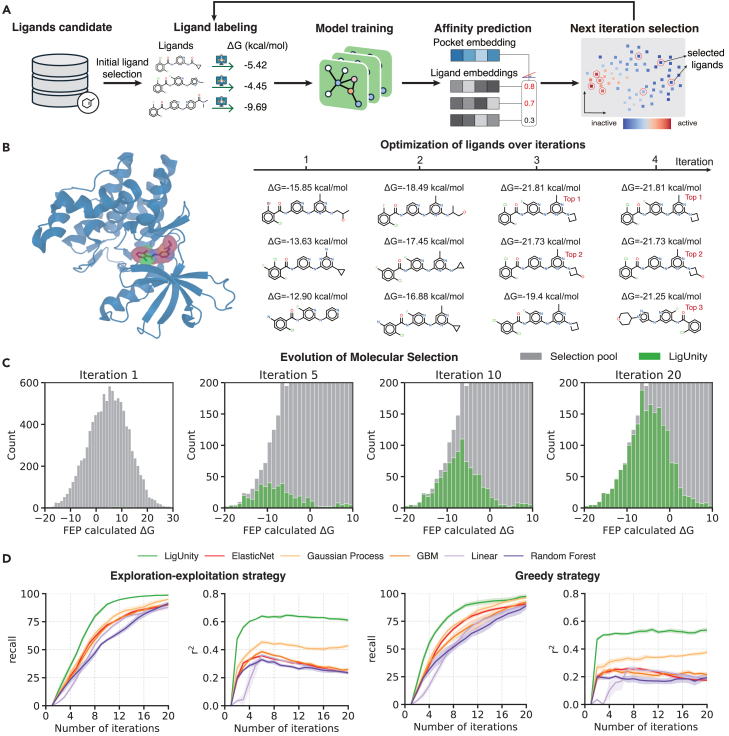
Evaluation of LigUnity in an active learning framework on TYK2 (A) An active learning framework based on LigUnity. (B) 3D structure for the TYK2 target (left, PDB: 4GIH) and the top ligands selected for iterations 1, 2, 3, and 4 (right) under the exploration-exploitation strategy; FEP-calculated binding free energies for each ligand are also shown. (C) Histogram plots showing the distribution of ΔG values of selected ligand for each iteration based on a greedy selection strategy. (D) Plots comparing LigUnity and competing methods on the TYK2 dataset in terms of top 2% recall and *r*^2^ when using the exploration-exploitation strategy (left) and greedy selection strategy (right).

To this end, we integrated LigUnity in an active learning framework, where we first trained the model using a small number of randomly selected ligands with known binding free energies. In each subsequent iteration, we selected a subset of unlabeled ligands and performed FEP calculations to determine their binding free energies. These newly labeled data points were then incorporated into the training set, and the model was retrained to refine its predictions (Figure 5A).

We compared all methods under two selection strategies: a greedy strategy and an exploration-exploitation strategy (see methods). We first found that LigUnity successfully identified the best active ligands within a few iterations (Figure 5B). The proportion of highly active ligands continually increased after each iteration (Figure 5C), demonstrating the effectiveness of the active learning framework in identifying highly active ligands. Moreover, we found that LigUnity consistently outperforms other competing methods under both greedy and exploration-exploitation strategies, achieving over 40% improvement in terms of *r*^2^ (Figure 5D). By comparing two different selection strategies, we found that the greedy strategy has a higher recall in early iterations by focusing on the most promising candidates (Figures 5D and S20). Interestingly, the exploration-exploitation strategy attains superior performance in later iterations, primarily because of its more diverse ligand sampling in early iterations that helps improve model accuracy. These findings demonstrate the broad applicability of LigUnity in drug design, particularly in identifying highly active ligands using limited experimental and computational resources.

## Discussion

We have presented LigUnity, a protein-ligand affinity foundation model for both virtual screening and hit-to-lead optimization. Our model learns a pocket-ligand shared space to capture structural and chemical complementarity between pockets and ligands, which is implemented through an integrated framework combining scaffold discrimination and pharmacophore ranking. For virtual screening, our experiments have shown that LigUnity outperformed 24 competing methods across three benchmarks (DUD-E, LIT-PCBA, and Dekois 2.0). For hit-to-lead optimization, our model achieves the best performance in two FEP benchmarks and demonstrates promising accuracy in split-by-time, split-by-scaffold, and split-by-unit settings. Moreover, by developing an active learning framework to simulate real-world lead optimization, LigUnity efficiently identifies optimal binding compounds with limited computational resources. Collectively, these results demonstrate the strong generalization ability and practical usability of LigUnity throughout the early-stage drug discovery pipeline, establishing our confidence in LigUnity as a versatile foundation model for computer-aided drug discovery.

Two main lines of work are related to LigUnity, including structure-free drug-target affinity (DTA) prediction methods and structure-based methods. Compared to DTA methods,^29^^,^^38^^,^^39^^,^^40^^,^^41^^,^^42^^,^^43^^,^^44^^,^^45^^,^^46^ which directly predict binding affinity using protein and ligand information, LigUnity presents three key differences. First, DTA methods are challenged by the small number of proteins with measured affinity (2,773 in BindingDB), whereas LigUnity incorporates fine-grained pocket information and leverages a comprehensive training set covering 53,406 pockets for 4,847 proteins, enabling robust performance on understudied proteins. Second, existing DTA methods perform direct regression of absolute affinity values, whereas LigUnity learns a pocket-ligand shared space using scaffold discrimination and pharmacophore ranking, reflected by the consistent performance improvement in our experiments. Moreover, our pharmacophore-ranking approach inherently functions as a normalization technique, focusing on relative ligand rankings rather than absolute affinity values, which mitigates the impact of experimental variability (e.g., pH and temperature) and assay-type differences (e.g., IC50 vs. K_D_). Third, DTA methods require intensive computation for each prediction, whereas LigUnity enables rapid screening by pre-computing and storing embeddings, leading to better efficiency for large-scale applications. Compared to structure-based methods^16^^,^^19^^,^^27^^,^^28^^,^^47^^,^^48^^,^^49^^,^^50^^,^^51^^,^^52^^,^^53^^,^^54^^,^^55^^,^^56^^,^^57^ that rely on 3D binding poses, LigUnity presents three improvements. First, structure-based methods are constrained by the scarcity of ligands with co-crystal structures. For example, only 7.5% of 0.57 million compounds with measured activities in BindingDB have crystal structures.^20^ In contrast, LigUnity is developed using a diverse training set of 0.45 million unique ligands, enabling effective generalization to novel chemical scaffolds. Second, structure-based methods require computationally expensive pose generation (approximately 1 min per compound using Glide), whereas LigUnity achieves competitive performance without the need for pose information, making it applicable to large-scale screening. Third, structure-based methods mostly rely on static protein conformations, whereas LigUnity implicitly accounts for pocket flexibility by leveraging multiple slightly varied structures for each pocket (averaging 10.96 pocket structures per assay). Furthermore, our approach offers significant flexibility for diverse scenarios. For novel targets with limited structural data, LigUnity can leverage predicted pockets from AlphaFold 3 or homology modeling. In cases where no experimental or predicted structures exist, we have also provided sequence-based variants of LigUnity.

Despite its promising performance in binding affinity prediction, LigUnity has two limitations that can be addressed in future work. First, LigUnity can only be applied to assays with known protein targets, limiting its applications to diverse assays without target information (e.g., phenotype assays). To expand the applicability of our model, we plan to incorporate additional modalities such as gene information in future work. Second, while LigUnity can successfully identify key substructures and residues and learn meaningful interaction patterns to improve its performance, its pose-free nature limits its ability to provide detailed mechanistic insights related to binding pose. Therefore, we plan to couple LigUnity with binding poses and develop a structure-based variant to enhance prediction accuracy and mechanistic interpretability, particularly for scenarios where sufficient computational resources are available to obtain precise binding poses.

## Methods

### Problem setting

LigUnity aims to predict binding affinity between given binding pockets and unmeasured ligands. Our training data are organized by assay, which is defined as the experimental procedure designed to evaluate the binding affinity of ligands against a specific protein target. Each assay has multiple measured ligands and only one target protein. A key consideration is that affinity values are only comparable within the same assay, and comparing affinity values from different assays targeting the same protein may not be reliable.^58^ This incomparability arises from variations in experimental conditions (e.g., cofactor concentration, pH, and temperature), assay formats (e.g., cell-based vs. target-based assays and different detection methods), and affinity measurements (e.g., IC50, Ki, and K_D_). Therefore, our method focuses on learning relative affinity rankings within each assay instead of absolute values.

Let A denote the set of assays. For each assay $A_{i}\in A$, $L_{i}$ denotes the set of tested ligands, and $v_{i}\left(l\right)$ denotes the affinity value of ligand $l\in L_{i}$. Each assay corresponds to one target protein, which may have multiple PDB structures available. We define $P_{i}$ as the set of protein pocket structures for assay $A_{i}$ and randomly sample one pocket structure from $P_{i}$ at each training step.

### Pocket and ligand encoder

In the pre-training stage, our pocket encoder and ligand encoder are initialized with Uni-Mol,^59^ an SE(3)-invariant graph transformer for 3D molecules, which is a structure-free method and does not rely on the binding pose as input. For the ligand branch, we first use the RDKit to generate 3D conformations for each ligand, which have been proven effective for various tasks,^60^ and then input them into the Uni-Mol ligand encoder to generate ligand embeddings. Similarly, for the pocket branch, we input the protein pocket conformation into the Uni-Mol pocket encoder to generate the protein embedding. For a pocket-ligand pair (p,l), their vector representations are obtained through

(Equation 1) $$\left(p,l\right)=\left(E_{p}\left(p\right),E_{l}\left(l\right)\right)\text{,}$$

where $E_{p}$ and $E_{l}$ denote the pocket and ligand encoders, respectively; p denotes the protein embedding; and l denotes the ligand embedding.

### Hierarchical affinity landscape navigation

In the pre-training stage, LigUnity learns a pocket-ligand shared space using two complementary strategies: scaffold discrimination and pharmacophore ranking. The scaffold discrimination approach captures the coarse-grained distinction between active and inactive pocket-ligand pairs by focusing on structural differences in the chemical scaffold, which aims to bring the embeddings of positive pocket-ligand pairs closer and push the negative pairs apart. The pharmacophore-ranking approach learns to align the embedding space with the fine-grained affinity ranking of measured ligands by focusing on the pharmacophore. These two approaches complement each other, and we jointly optimize them to make the model suitable for both virtual screening and hit-to-lead optimization scenarios.

During inference, the model only needs to take 3D protein pockets and 3D ligand conformations (generated by the RDKit) as input. It then calculates the pocket and ligand embeddings and outputs their cosine similarity as the predicted affinity score.^13^ This differs from most previous ML affinity prediction models, which typically model affinity prediction as a regression task and use either dual-tower architectures (structure-free methods)^29^^,^^42^^,^^44^ or require binding poses (structure-based methods)^27^^,^^28^ as input. Once the pocket and ligand embeddings are pre-computed, LigUnity enables fast screening by similarity-based comparison in joint embedding space, achieving six magnitudes of speedup compared to docking methods.^13^

#### Pharmacophore ranking

Pharmacophore ranking is an approach that prioritizes active compounds based on their binding potential and structural nuances. We implement pharmacophore ranking through listwise learning. Unlike pointwise approaches that predict absolute scores or pairwise approaches that compare item pairs, listwise ranking considers the entire ranked list simultaneously, making it particularly suitable for problems where the relative ordering matters more than absolute values. This approach allows us to capture subtle structural differences between ligands that share similar scaffolds but exhibit varying binding affinities to the same pocket. We adopt the Plackett-Luce model,^61^ a statistical model specifically designed for ranking data. The fundamental idea of the Plackett-Luce model is to assign each item a positive “strength” parameter, which determines the probability of the item being selected. In our context, the strength parameter $\theta_{i}$ for each ligand is computed as the exponential of its pocket-ligand similarity score: $\theta_{i}=\exp\left(\text{sim}\left(p,l_{i}\right)\right)$. For a set of items with strength parameters $\left\{\theta_{1},\ldots ,\theta_{m}\right\}$, the probability of selecting item i is defined as

$$P\left(i\;\text{is}\;\text{chosen}\right)=\frac{\theta_{i}}{\sum_{j=1}^{m}\theta_{j}}\text{.}$$

The Plackett-Luce model treats ranking as a sequential selection process. Given n ligands to rank, the model first selects the top-ranked ligands from all candidates using the above probability formula. Then, it selects the second-ranked ligand from the remaining n−1 candidates, again using the same probability formula but only considering the remaining ligands. This process continues until all ligands are ranked. The probability of observing a complete ranking sequence π is the product of these conditional probabilities at each selection step:

$$P\left(\pi \right)=\prod_{k=1}^{n}\frac{\theta_{\pi \left(k\right)}}{\sum_{j=k}^{n}\theta_{\pi \left(j\right)}}\text{,}$$

where π(k) denotes the index of the ligand at position k in the ranking. The model parameters are optimized by minimizing the negative log likelihood of observing the true ranking based on the measured affinity values. This framework naturally handles varying numbers of ligands across different assays and provides a probabilistic interpretation of the ranking process.

Given this probabilistic framework, we can define the ranking loss for assay $A_{i}$ as the weighted negative log likelihood of the observed ranking probability. For each assay $A_{i}\in A$, we order ligands according to their measured affinity values. At the k-th step of the selection process, considering all ligands with affinity values no greater than that of the k-th ranked ligand, we define the selection probability for the k-th ranked ligand as

(Equation 2) $$\pi \left(l_{k}|L_{i}^{\leq k},p\right)=\frac{\exp\left(\text{sim}\left(p,l\right)/\tau \right)}{\sum_{j\in L_{i}^{\leq k}}\exp\left(\text{sim}\left(p,l_{j}\right)/\tau \right)}\text{,}$$

(Equation 3) $$\text{where}\;p=E_{p}\left(p\right),\text{and}\;l=E_{l}\left(l\right)\text{,}$$

where $p\in P_{i}$ is the 3D structure of the pocket tested in the assay $A_{i}$; $l_{k}$ is the k-th ranked ligand; $L_{i}^{\leq k}$ represents the set of ligands with affinity values no greater than $v_{i}\left(l_{k}\right)$; τ is a temperature hyper-parameter; and sim(p,l) denotes the cosine similarity between protein and ligand embedding vectors.

Based on these stepwise selection probabilities, we compute the ranking loss as the weighted sum of the negative log probabilities over all selection steps:

(Equation 4) $$L_{i}^{r}=-\sum_{k=1}^{\left|L_{i}\right|}\mu_{k}\;\log\;\pi \left(l_{k}|L_{i}^{\leq k},p\right)\text{,}$$

(Equation 5) $$\text{where}\;\mu_{k}=\frac{1}{\sqrt{\left|L_{i}\right|}\log\left(k+1\right)}\text{,}$$

where $\mu_{k}$ is a decay factor with two components: 1/log(k+1) prioritizes top-ranked molecules and $1/\sqrt{\left|L_{i}\right|}$ prevents assays with many ligands from dominating the training loss. We also experimented with $1/\left|L_{i}\right|$ and constant scaling terms and empirically found that $1/\sqrt{\left|L_{i}\right|}$ yields the best performance.

#### Scaffold discrimination

Scaffold discrimination, implemented through contrastive learning, aims to maximize the similarity between active pocket-ligand pairs while minimizing the similarity between inactive pairs. This approach enables the model to learn global structure-activity relationships across diverse chemical scaffolds, providing a foundation for broadly distinguishing active from inactive compounds. Following Gao et al.,^13^ we formulate our contrastive loss using in-batch softmax, which consists of two components: the pocket-to-ligand loss $L_{i}^{p\to l}$ and the ligand-to-pocket loss $L_{i}^{l\to p}$:

(Equation 6) $$L_{i}^{p\to l}=-\frac{1}{\left|L_{i}^{a}\right|}\sum_{k\in L_{i}}\log\frac{\exp\left(\frac{\text{sim}\left(p,l_{k}\right)}{\tau }\right)}{\sum_{m\in L_{batch}}\exp\left(\frac{\text{sim}\left(p,l_{m}\right)}{\tau }\right)},\text{and}$$

(Equation 7) $$L_{i}^{l\to p}=-\frac{1}{\left|L_{i}^{a}\right|}\sum_{k\in L_{i}}\log\frac{\exp\left(\frac{\text{sim}\left(p,l_{k}\right)}{\tau }\right)}{\sum_{n\in P_{batch}}\;\exp\left(\frac{\text{sim}\left(p_{n},l_{k}\right)}{\tau }\right)}\text{,}$$

(Equation 8) $$\text{where}\;p=E_{p}\left(p\right)\;\text{and}\;l=E_{l}\left(l\right)\text{,}$$

where $p\in P_{i}$ is a 3D structure of the pocket tested in the assay $A_{i}$; $L_{batch}$ and $P_{batch}$ denote the set of ligands and pockets sampled in this training batch, respectively; τ is a temperature hyper-parameter, and $L_{i}^{a}$ denotes the set of active ligands tested in the assay $A_{i}$.

Building on this formulation, we define how positive and negative pairs are identified in our framework: pocket-ligand pairs with experimentally measured affinity (≤10μmol) are treated as positive pairs following Mayr et al.,^62^ while pairs without measured affinity values serve as negative pairs. Although the boundary between active and inactive ligands varies across different protein targets, our framework naturally addresses this issue by learning the relative affinity among ligands rather than relying solely on binary active/inactive labels. However, this in-batch negative sampling may introduce potentially harmful false negative pocket-ligand pairs. To mitigate this, we devised a simple strategy to identify potentially active pocket-ligand pairs. Specifically, pocket-ligand pairs formed between assay A and assay B are excluded from negative sampling if the protein targets of the two assays share the same UniProt ID. This exclusion is based on the observation that protein pockets with the same UniProt ID are likely to share overlapping active ligands.

The total contrastive loss is defined as

(Equation 9) $$L^{c}=\frac{1}{\left|A\right|}\sum_{i\in A}\gamma_{i}\left(L_{i}^{p\to l}+L_{i}^{l\to p}\right)\text{,}$$

where $\gamma_{i}=1/\sqrt{\left|L_{i}\right|}$ prevents assays with many ligands from dominating the training loss.

#### Pre-training objective

To optimize both virtual screening and hit-to-lead optimization, LigUnity is pre-trained with a combined loss that integrates scaffold discrimination and pharmacophore ranking:

(Equation 10) $$L=L^{c}+L^{r}\text{.}$$

Collectively, this hierarchical approach enables LigUnity to navigate the affinity landscape by first broadly discriminating active scaffolds from inactive ones and then precisely ranking ligands based on their pharmacophoric features, supporting its effectiveness in both virtual screening and hit-to-lead optimization tasks. We demonstrated that jointly optimizing these two tasks can make each help the other, which is shown in our ablation studies (Figures S13 and S16).

### H-GNN

Based on the observation that structurally similar pockets tend to have similar interaction patterns with ligands, we hypothesize that integrating information from existing large-scale pocket-ligand databases should improve the performance of LigUnity. To this end, we propose a H-GNN model to refine pocket embeddings by incorporating information from a large-scale pocket-ligand knowledge graph.

Our H-GNN model consists of two main components: a pocket-pocket aggregator and a pocket-ligand aggregator. This model receives a directed knowledge graph $G=\left(V_{p},V_{l},E_{l\to p},E_{p\to p}\right)$ of pockets and ligands as input, where $V_{p}=\left\{p_{i}\right\}\;\text{and}\;V_{l}=\left\{l_{i}\right\}$ are two sets of nodes representing the pockets and ligands, respectively; $E_{l\to p}$ is the ligand-to-pocket edge set, and $E_{p\to p}$ is the pocket-to-pocket edge set. For ligand-to-pocket edges, we add an edge from a ligand to a pocket if the ligand is active for the protein pocket with experimentally measured affinity. For pocket-to-pocket edges, we add edges between pockets based on sequence alignment scores, which are computed using scikit-bio with the BLOSUM50 substitution matrix. We only add edges between pockets with high alignment scores. Specifically, if the alignment score between pocket A and pocket B is larger than half of the self-alignment score of pocket A, we add an edge from pocket B to pocket A.

We constructed this large-scale pocket-ligand knowledge graph from our pre-training data. The graph contains 53,406 pocket nodes, 0.45 million ligand nodes, 0.83 million ligand-to-pocket edges, and 16 million pocket-to-pocket edges. During the virtual screening, the H-GNN model refines the embedding of the query protein pocket by incorporating information from the heterogeneous graph: $p_{k}^{*}=H-\text{GNN}\left(p_{k}\right)$. We then use this refined pocket embedding $p_{k}^{*}$ to retrieve hit ligands in the candidate ligand embedding space.

#### Pocket-pocket aggregator

We first use a pocket-pocket aggregator to incorporate information from similar pockets, based on the observation that structure-similar pockets often exhibit similar interaction patterns with ligands. The aggregation function is defined as

(Equation 11) $$p_{k}^{p}={\text{AGG}}_{p}\left(p_{i}|\left(i,k\right)\in E_{p\to p}\right)\text{,}$$

where $p_{k}^{p}$ is the refined embedding of the k-th pocket $p_{k}$ that aggregates information from its neighboring pockets; ${\text{AGG}}_{p}$ is implemented using an attention mechanism, which is defined as follows:

(Equation 12) $${\text{AGG}}_{p}\left(p_{i}|\left(i,k\right)\in E_{p\to p}\right)=\sum_{\left(i,k\right)\in E_{p\to p}}\alpha_{i}p_{i}\text{,}$$

(Equation 13) $$\text{where}\;\alpha_{i}=\frac{\exp\left({\tilde{\alpha }}_{i}\right)}{\sum_{\left(n,k\right)\in E_{p\to p}}\;\exp\left({\tilde{\alpha }}_{n}\right)}\text{,}$$

(Equation 14) $$\text{where}\;{\tilde{\alpha }}_{i}=W_{2}^{T}\cdot \text{ReLU}\left(W_{1}\cdot \left[p_{i}|p_{k}\right]+b_{1}\right)\text{,}$$

where $\alpha_{i}$ denotes the normalized attention weight for the neighboring pocket i; $\left[p_{i}|p_{k}\right]$ denotes the concatenation of vectors $p_{i}$ and $p_{k}$; and $W_{1}\in R^{d_{h}\times 2d}$, $b_{1}\in R^{d_{h}}$, and $W_{2}\in R^{d_{h}\times 1}$ are three learnable parameters, where d is the hidden dimension of pocket embeddings and $d_{h}$ is the hidden dimension of the attention network.

#### Pocket-ligand aggregator

After aggregating information from similar pockets, we next utilize the pocket-ligand aggregator to aggregate information from 2-hop ligand neighbors, based on the observation that structure-similar pockets should have structure-similar active ligands. Let $N_{k}^{2}$ denote the set of 2-hop ligand neighbors of pocket k:

(Equation 15) $$N_{k}^{2}=\left\{j|\exists i:\left(j,i\right)\in E_{l\to p}\wedge \left(i,k\right)\in E_{p\to p}\right\}\text{;}$$

the aggregation function is defined as

(Equation 16) $$p_{k}^{l}={\text{AGG}}_{l}\left(\Phi \left(\left[l_{j}|r_{kj}\right]\right)|j\in N_{k}^{2}\right)\text{,}$$

where $p_{k}^{l}$ is the refined embedding of the k-th pocket that aggregates information from the active ligands of its neighbor pockets; $l_{j}$ is the ligand embedding of the j-th ligand; $r_{kj}$ is a relation embedding derived from the similarity score between the k-th pocket and its intermediate neighbor (the i-th pocket) where the j-th ligand is active for it; and Φ is a 2-layer multilayer perceptron (MLP) with the hidden size of 2d. Similar to the pocket-pocket aggregator, ${\text{AGG}}_{l}$ is also implemented using an attention mechanism with different learnable parameters.

#### Refined pocket embedding

Finally, we combine the original pocket embedding with the refined embeddings from both aggregators to obtain the final refined pocket embedding:

(Equation 17) $$p_{k}^{*}=\gamma_{1}p_{k}^{l}+\gamma_{2}p_{k}^{p}+\left(1-\gamma_{1}-\gamma_{2}\right)p_{k}\text{,}$$

where $\gamma_{1},\gamma_{2}\in \left[0,1\right]$ and $\gamma_{1}+\gamma_{2}\leq 1$ are parameters that control the contribution of each component; this weighted combination allows the model to adaptively balance information from different sources: similar pockets, their active ligands, and the pocket’s intrinsic features. Collectively, our proposed H-GNN integrates information from both similar pockets and their active ligands, leveraging the observation that structure-similar binding pockets tend to have similar active ligands, to enhance the performance of LigUnity in finding active ligands for the query protein pocket. The effectiveness of H-GNN is shown in our ablation studies (Figures S11–S13).

### Competing methods

In this paper, we compared LigUnity with three types of competing methods: (1) docking score methods including Glide (SP and XP),^25^ Vina,^26^ AutoDock4,^63^ Vinardo,^64^ Gold,^65^ Surflex,^66^ and Flexx^67^; (2) structure-based ML methods that take a protein-ligand complex structure as input, including NNscore 2.0,^48^ RFscore v.4,^49^ $\Delta_{\text{Vina}}\text{RF,}$^50^ $\Delta_{\text{Lin}\_F9}\text{XGB,}$^52^ RTMScore,^27^ Genscore,^16^ Pafnucy,^53^ OnionNet,^54^ Planet,^56^ PIGNet,^55^ EquiScore,^19^ Kdeep,^68^ TANKBind,^69^ 3D-GNN,^55^ PBCNet,^15^ Denvis,^28^ and GNINA^57^; and (3) the structure-free methods DeepDTA,^29^ BIND,^70^ DrugCLIP,^13^ Pocket-DTA (the regression-based version of LigUnity), and its protein sequence-based version Sequence-DTA. For experiments on the Merck and JACS benchmarks, we additionally compared LigUnity with physics-based binding free energy calculation methods including Prime-MM/GBSA^32^ and FEP+.^9^ For active learning experiments on the TYK2 dataset, we compared five ML methods, including ElasticNet,^71^ Gaussian process,^72^^,^^73^ gradient boosting machine (GBM),^74^ linear regression, and random forest,^75^ and these methods take 2,048D ECFP4 fingerprints as input.

## Resource availability

### Lead contact

Requests for further information and resources should be directed to and will be fulfilled by the lead contact, Sheng Wang (swang@cs.washington.edu).

### Materials availability

This study did not generate new unique reagents.

### Data and code availability

• The training dataset has been deposited at Figshare: https://doi.org/10.6084/m9.figshare.27966819.^76^ The PocketAffDB with protein and pocket PDB structures has been deposited at Figshare: https://doi.org/10.6084/m9.figshare.29379161.^77^ The processed Dekois 2.0 benchmark dataset has been deposited at Figshare: https://doi.org/10.6084/m9.figshare.27967422.^78^
• This paper collected training data from existing, publicly available datasets, accessible at https://doi.org/10.6019/CHEMBL.database.34^21^ (ChEMBL34 dataset) and https://www.bindingdb.org^20^ (BindingDB dataset v.2024m5). This paper conducted experiments on existing, publicly available datasets, accessible at https://doi.org/10.1007/s10822-022-00478-x^35^ (PDE10A dataset), https://doi.org/10.1016/j.ailsci.2022.100050^36^ (TYK2 binding free energy dataset), and https://doi.org/10.1038/s42004-023-01019-9^79^ (JACS^9^ and Merck^31^ benchmarks).
• All original code has been deposited at GitHub and is publicly available at https://github.com/IDEA-XL/LigUnity^80^ as of the date of publication.
• Any additional information required to reanalyze the data reported in this paper is available from the lead contact upon request.

## Acknowledgments

We’d like to express our gratitude to Dr. Zequn Liu, Dr. Kangjie Zheng, and Dr. Junwei Yang from Peking University for their valuable discussions and assistance in this work. We are also grateful to Dr. Michael K. Gilson and Dr. Tiqing Liu from the University of California, San Diego (UCSD), for their expert guidance on data curation from BindingDB. B.F., Z.L., H.L., H.C., Y.L., and L.Z. are supported by the Hetao Shenzhen-Hong Kong Science and Technology Innovation Cooperation Zone, Shenzhen, China under grant no. HTHZQSWS-KCCYB-2023052.

## Author contributions

B.F., Z.L., Y.L., L.Z., and S.W. contributed to the conception and design of the work. B.F. and Z.L. contributed to the data curation. B.F. and Y.L. contributed to the technical implementation. B.F., Z.L., H.L., Y.L., L.Z., and S.W. contributed to technical discussions. B.F., Z.L., M.Y., J.Z., and S.W. contributed to the evaluation framework used in the study. All authors contributed to the drafting and revision of the manuscript.

## Declaration of interests

The authors declare no competing interests.

## Supplemental information

Document S1. Figures S1–S21, Tables S1–S4, and supplemental methods

Document S2. Article plus supplemental information
