# Supplementary material for: Hierarchical affinity landscape navigation through learning a shared pocket-ligand space
Source: Patterns (N Y). 2025 Sep 17;6(10):101371. doi: 10.1016/j.patter.2025.101371 (PMC12546767; doi:10.1016/j.patter.2025.101371)
Supplement: Document S1. Figures S1–S21, Tables S1–S4, and supplemental methods [file mmc1.pdf]

Patterns, Volume *6*

Supplemental information

Hierarchical affinity landscape navigation

through learning a shared pocket-ligand space

Bin Feng, Zijing Liu, Hao Li, Mingjun Yang, Junjie Zou, He Cao, Yu Li, Lei Zhang, and Sheng Wang

Supplementary Information

Contents

1 Implementation details **2**

2 Data curation **2**

2.1 Organizing affinities into assays . . . 2

2.2 Assay-guided pocket matching . . . 3

2.3 Removing duplicate assays . . . 3

3 Experiments on virtual screening benchmarks **3**

4 Experiments on FEP benchmarks **4**

5 Experiments under diverse settings **5**

6 Active learning experiment on the TYK2 target **5**

References **19**

1 Implementation details

The details of our training strategy are listed as follows. In the pre-training stage, we trained LigUnity for 50 epochs, and we used the averaged parameter of the last 10 epochs for testing. We warmed up the learning rate for the first 6% step and adopted a polynomial decay learning rate scheduler afterward. We used the Adam optimizer with a maximum learning rate of 0.0001. For each training iteration, we sampled 24 assays for computing loss. For each assay, we randomly sampled one pocket structure and a maximum of 16 measured ligands coming from the assay, making sure that their experimental affinity values were comparable to each other. We used listwise ranking for measured ligands that belonged to the same assay and used contrastive loss between the whole sampled ligands and pockets in this batch.

For Heterogeneous-GNN (H-GNN), we trained it separately from the pocket and ligand encoder. We trained H-GNN for 10 epochs and used the last epoch for testing. We adopted the constant learning rate scheduler with a learning rate of 0.001. For each training iteration, we randomly sampled 128 assays, and we randomly selected one pocket structure and one active ligand for each assay. As the large-scale nature of the pocket-ligand knowledge graph makes it hard to fine-tune H-GNN on a few measured ligands, we adopted H-GNN only for virtual screening tasks and used the contrastive loss between the whole ligands and pockets sampled in this batch for training.

For LigUnity(seq), the protein-based version of LigUnity, we used ESM2[1] 35M for encoding the protein sequence in replacement of the 3D pocket encoder, and we updated the parameters of ESM2 during training. Furthermore, we also attempted to use the larger ESM2 3B model with a frozen gradient but observed a drop in test performance. To fully make use of the pocket and protein information, the results of LigUnity are reported as the ensemble of the pocket-based model and the protein-based model, except for the experimental setting where training proteins with >30% sequence similarity are removed. This exception is made because protein-based models typically show limited generalization ability to unseen protein families with low sequence similarity.

We also trained Pocket-DTA and Sequence-DTA, the regression-based version of LigUnity and LigUnity(seq) respectively, to compare different protein-ligand affinity prediction strategies. In the pre-training stage, we used mean square loss and margin loss for training. First, for pocket-ligand pairs with experimental affinities, we used the mean square loss for training which is defined as the mean square error between experimental affinity and predicted affinity $\left(L_{SE} = (y_i - \hat{y}i)^2\right)$. Second, to enable Pocket-DTA to distinguish between active and inactive ligands, we introduced in-batch margin loss. For unmeasured in-batch pocket-ligand pairs, we consider them to be inactive and employ margin loss to penalize them $\left(L_{Margin} = \max(0, \hat{y} - (y_{min} - m))^2\right)$, where y_{min} is the minimum known activity known to the pocket and m is a margin hyper-parameter. We set the margin to 2.0 after a grid search on the validation set.

2 Data curation

The purpose of our data curation is to collect high-quality pocket-ligand binding affinity data and organize them by assays. We developed a curation pipeline based on the BindingDB[2] and ChEMBL[3] datasets. ChEMBL is a large-scale bioactivity database that integrates experimental data from scientific publications, patents, and other datasets. It covers various aspects of drug discovery and development, containing 2.5 million unique compounds and 19 million experimentally measured activities. BindingDB is another large-scale database that focuses specifically on protein-ligand binding affinities. It is curated mainly from scientific publications and US patents, containing 0.5 million compounds, 1.1 million measured affinities, and 2,773 protein targets.

Our data curation pipeline consists of three stages: organizing affinities into assays, assay-guided pocket matching, and removing duplicate assays. In this study, we used the 34 version of ChEMBL and the 2024m5 version of BindingDB downloaded from their official websites. The details of our curation pipeline are as follows:

2.1 Organizing affinities into assays

Considering that the affinities from different assays are incompatible due to varying experimental conditions (e.g., cofactor concentration, pH, temperature), assay formats (e.g., cell-based vs. target-based assays, and different detection methods), and affinity measurements (e.g., IC50, Ki, Kd), we first organized the experimental affinities into assays. For ChEMBL, we collected all assays with “Binding” type and known protein targets, then we grouped affinity data by ChEMBL assay ID. We only retained affinity data with molar concentration units (e.g., nmol) or density units (e.g., μg/ml) to ensure data quality, as the exact meaning of other units (e.g., % or no unit) was difficult to determine. This filtering step removed 33.1% of the activity data in ChEMBL. For BindingDB, we grouped the affinity data with the same protein target, affinity measurement, source document, and assay description into an assay following Actfound[4], ensuring that affinity values within the same assay were comparable. We also removed inorganic ligands and large molecular ligands (molecular weights > 1,000). Finally, we retained assays with more than 5 affinity values for further curation. We collected 69,872 and 25,902 assays from ChEMBL and BindingDB respectively, with each assay having one corresponding protein target.

2.2 Assay-guided pocket matching

Based on the observation that most assays are designed for a specific binding site of interest, we hypothesize that ligands in an assay should bind to the same pocket of a protein. Therefore, once we identified a binding pocket for one ligand in an assay, we assumed that other ligands in the same assay would bind to the identical pocket. For each assay, we identified binding pockets in the PDB (Protein Data Bank)[5] using two criteria: protein sequence similarity and ligand Tanimoto similarity. First, we searched for protein-ligand complex structures whose protein sequence similarity with the protein studied in this assay is above 40%, as proteins with such similarity typically share high structural and functional homology[6]. Then, we calculated the Tanimoto similarity between the crystal ligands in these PDB structures and the assay ligands. If the ECFP4 Tanimoto similarity exceeded 70%, we collected the binding pocket of the crystal ligand as the candidate pockets for this assay, as ligands with such high similarity are likely to bind to the same pocket in a protein[7]. We have trained model on dataset curated with 100% Tanimoto similarity threshold and observed a performance decrease (**Table S4**).

To discard biologically irrelevant pockets that have weak binding interactions with the ligand (i.e., those having a small number of binding residues with ligands), we used the processed binding pocket database provided by BioLip[8]. After this procedure, we collected 25,883 and 16,863 assays from ChEMBL and BindingDB respectively. For each assay, there are 7.19 identified pockets on average. Although the identified pockets for the same assay have different PDB IDs, they mostly belong to the same binding site. To verify this, we aligned complex PDB structures retrieved for each assay and computed the maximum distance between the centers of all pockets. We identified 1,229 and 802 assays whose maximum distance between retrieved pockets exceeded 10 Å for ChEMBL and BindingDB respectively, accounting for only 4.8% of the total assays. We have trained model on assays without multiple pockets (>10Å) and observed a performance decrease (**Table S4**).

2.3 Removing duplicate assays

As ChEMBL and BindingDB share common data sources including scientific literature and patents, there could be many overlapping assays between these two datasets. Additionally, there are some repeating assays within the ChEMBL or BindingDB database as well. To reduce the negative impact of data redundancy on pre-training, we detected and removed identical assays following pQSAR-ChEMBL[9]. Specifically, if the Pearson correlation (ρ_p) of affinities between the shared ligands of two assays exceeds a threshold of 0.95, we considered those two assays to be identical. After this stage, we detected and removed 15,664 repeating assays, ending up with 26,748 non-repeating assays curated from ChEMBL and BindingDB.

After the above curation procedures, we collected 26,748 assays with 428,767 unique ligands and 36,662 unique pockets from ChEMBL, BindingDB, and PDB, covering 2,196 unique proteins. Each assay has on average 30.29 measured ligands and 10.96 pockets from PDB. Our curation process has created a large-scale pocket-ligand affinity dataset while maintaining the quality of the curated protein binding pockets, which is supported by the small fraction of assays with distinct pockets.

However, due to the limited number of unique proteins in the original binding database (2,773 in BindingDB), models trained solely using these data might result in poor generalization on unseen proteins. To address this limitation, we further enlarged our curated dataset by utilizing the protein-ligand complex data from PDB[10], which includes diverse proteins. For each protein-ligand complex with crystal structure, we considered the ligand to be active following previous methods[11,12] and created one assay that contained only one protein pocket and one active ligand. We did not merge ligands binding to the same protein into one assay, as we could not determine the relative affinity ranking between these ligands. To avoid the negative impact of the low-quality complex structure data in PDB (those with low resolution, inactive ligands, or biologically irrelevant ligands), we directly used the PDBBind v2020[13] dataset for training, following previous methods[11,12]. The PDBBind dataset contains 3,889 unique proteins, and the number of unique proteins and pockets in our curated pre-training data finally reached 4,847 and 53,406 respectively.

3 Experiments on virtual screening benchmarks

For virtual screening, we evaluated our method on three benchmark datasets (DUD-E[14], Dekois[15], and LIT-PCBA[16]), all of which have confirmed binding sites. For DUD-E and LIT-PCBA benchmarks, we used the processed version from DrugCLIP's repository[12]. For the Dekois 2.0 benchmark, we downloaded it from the official website and extracted residues within 6 Å distance from the crystal ligand to form the binding pocket. To prevent data leakage from overlapping protein targets between our curated pre-training data and test benchmarks, we excluded proteins that appear in the three benchmarks from our pre-training data and used the model trained on this processed dataset for testing.

The DUD-E benchmark contains 102 targets, with an average of 224.4 active ligands per target. For each active ligand, there are approximately 50 decoys with similar physicochemical properties, with over 99% of decoys not experimentally verified. The Dekois 2.0 benchmark follows a similar construction approach, containing 81 targets, each with 30 active ligands and 40 generated decoys per active ligand. The LIT-PCBA benchmark has 15 targets with an average of 533.3 active ligands per target. It contains 2.64 million inactive ligands with an active-to-inactive ratio of 1:1000, better reflecting real-world drug design scenarios. Additionally, all active and inactive ligands in LIT-PCBA have been experimentally verified. In contrast, DUD-E

and Dekois 2.0 benchmarks exclude decoys with similar structures to avoid potential active ligands, which may make them relatively easy for ML models. For DUD-E, the results of Denvis-G, Denvis-R, DeepDTA, Gold, Surflex, Flexx, Vina, and GNINA are from Krasoulis et al.[17]; the results of other structure-based methods are from Cao et al.[18]. For Dekois 2.0, the results of all docking methods and structure-based methods are from Cao et al.[18]. For LIT-PCBA, the evaluation results of Denvis-G, Denvis-R, GNINA, and DeepDTA are from Krasoulis et al.[17]; the results of GenScore and Glide SP are from Shen et al.[19]

Following previous works[11,12,17], we evaluated methods using three metrics: Enrichment Factor (EF), Area Under the Receiver Operating Characteristic curve (AU-ROC), and Boltzmann-Enhanced Discrimination of ROC (BEDROC). EF quantifies a model's ability to identify actives among top-ranked ligands, calculated as the proportion of actives in top-ranked ligands (e.g., top 1%) divided by the proportion of actives in all candidate ligands. We use EF 1% for evaluation, which ranges from 0 to 100. AU-ROC assesses a model's ability to correctly rank active ligands before inactive ligands and is defined as the area under the plot of true positive rate against false positive rate. However, since AU-ROC treats all ranked ligands equally, it may not be ideal for virtual screening tasks where only top-ranked ligands undergo costly experimental verification. To address this limitation, we employ BEDROC, which assigns higher weights to top-ranked ligands. Following previous works[11,12,17], we set the α parameter to 80.5 to balance the evaluation of top-ranked ligands and overall ranking performance. AU-ROC and BEDROC both range from 0 to 1. For all metrics, higher values indicate better performance.

To further assess LigUnity's generalizability to unseen proteins, we conducted experiments on unseen protein families. We excluded training proteins with more than 80% and 30% sequence similarities to any test protein on the three benchmarks; then we used the model trained on this processed dataset for evaluation. We used cd-hit to compute protein sequence similarity, which is defined as the number of identical residues after alignment.

4 Experiments on FEP benchmarks

To test the performance of LigUnity on hit-to-lead optimization scenarios, we evaluated all methods on two FEP calculation benchmarks (JACS[20] and Merck[21]), all of which have confirmed binding sites. The JACS and Merck benchmark datasets, along with the results of FEP+(OPLS4)[20] calculated using the state-of-the-art OPLS4[22] force field, were obtained from the public repository `https://github.com/schrodinger/public_binding_free_energy_benchmark`[23]. These FEP benchmarks contain 16 targets (assays), with each target having an average of 29 experimentally measured ligands. We extracted residues within a 6 Å distance from the crystal ligand as the binding pocket. For experiments on these FEP calculation benchmarks, we used Spearman correlation (ρ_s) and $r^2 = \max(0, \rho_p)^2$ for evaluation.

For zero-shot evaluation on FEP benchmarks, we directly applied LigUnity for inference without fine-tuning. To mitigate potential data leakage in the pre-training data, we removed assays that were similar to assays in test benchmarks from the pre-training data. Following previous methods[4,9], we defined two assays as identical if the affinities of overlapping ligands reached a Pearson's correlation of 0.95. We also removed all ligands from the pre-training data that existed in FEP benchmarks to ensure a zero-shot setting. To reduce variability in evaluation metrics due to the limited number of targets in the FEP benchmarks, we trained five models with different random seeds for all our implemented methods (LigUnity, LigUnity(seq), Pocket-DTA, and Sequence-DTA), and reported the average evaluation metrics. The evaluation results (ρ_s) of all computational methods and structure-based methods on the Merck benchmark are from Shen et al.[19]

For few-shot experiments, the fine-tuning ligands for each test assay were randomly sampled with a uniform distribution, and we evaluated LigUnity's performance on the remaining ligands. We evaluated all methods with varying proportions of fine-tuning ligands ranging from 20% to 80%. Following previous methods[4], we repeated the test experiments 40 times. For all our implemented methods (LigUnity, LigUnity(seq), Pocket-DTA, and Sequence-DTA) and DrugCLIP, we performed 5 steps of fine-tuning on the fine-tuning ligands using the same learning rate as in pre-training, and the final evaluation metrics were obtained by averaging the model's predictions on test ligands from all steps.

To study the generalization ability of LigUnity on unseen chemical scaffolds and proteins, we investigated three increasingly challenging settings: (1) no similar ligands setting, where we excluded training ligands with more than 50% ECFP4 Tanimoto similarity to any test ligand, the similarity is computed using 2048-dimensional ECFP4 fingerprints generated by RDKit; (2) no similar proteins setting, where we excluded training proteins with more than 30% sequence similarity to any test protein (threshold set following AlphaFold3[6]), and we used cd-hit to compute protein sequence similarity defined as the number of identical residues after alignment; (3) no similar ligands and proteins setting, where we applied both criteria from the first two settings.

For calculating the importance score of each ligand atom and protein residue, we replaced atomic features with padding tokens and measured the resulting decrease in affinity prediction, where larger decreases indicated higher importance of the masked atoms. In this study, we used the model trained on the "no same ligands" setting, and we used the PDB structure 5EHR and its crystal ligand 5OD for the case study.

5 Experiments under diverse settings

After evaluation on FEP benchmarks, we next sought to evaluate LigUnity on assays from ChEMBL[3] and BindingDB[2], which contain diverse assays covering various aspects of assay types. We evaluated all methods under two assay split settings: split-by-time and split-by-unit. Under each assay split setting, we also investigated two ligand split settings: uniformly split at random and split-by-scaffold.

For the split-by-time setting, we first pre-trained all models using assays released before March 2019, and then conducted testing on assays released after March 2019. To evaluate generalization capability on truly unseen proteins, we only evaluated on assays containing previously unseen proteins. This test set comprised 161 assays, with an average of 48.6 ligands per assay.

For the split-by-unit setting, we collected 65 assays with unit "%" and assay type "Activity" from ChEMBL for testing, differing from our pre-training assays that have molar concentration units (e.g., nmol) or density units (e.g., μg/ml). This test set contains 65 assays in total, with an average of 30.5 ligands per assay. In this setting, we used the model trained in the split-by-time setting for evaluation.

For few-shot evaluation on both split-by-time and split-by-unit settings, we evaluated all methods with varying proportions of fine-tuning ligands ranging from 4 to 16. We repeated the test experiments 10 times. For all our implemented methods (LigUnity, LigUnity(seq), Pocket-DTA, and Sequence-DTA) and DrugCLIP, we performed 10 steps of fine-tuning on the fine-tuning ligands using the same learning rate as in pre-training, and the final evaluation metrics were obtained by averaging the model's predictions on test ligands from the last 5 steps (steps 6-10). For the split-by-scaffold setting on each test assay, we first generated Bemis-Murcko chemical scaffolds for all ligands using RDKit; then we iteratively selected molecules sharing the same scaffold as fine-tuning ligands until reaching the preset number, while using the remaining molecules for testing.

For evaluation on the PDE10A target, we used the dataset released by Roche Ltd. in 2022[24]. This dataset comprises 1,162 ligands with experimentally measured binding affinities and corresponding experimental timestamps. To simulate the realistic drug development scenarios, we followed the split-by-time setting at both assay and ligand levels (**Figure 4e**). At the assay splitting level, we used the model pre-trained on assays published before March 2019, while the PDE10A dataset was released in 2022. At the ligand splitting level, we used 20% of the ligands measured earlier for fine-tuning and the remaining 80% of ligands measured later for testing. As the number of fine-tuning ligands is more than 100, we performed 20 steps of fine-tuning, and the final evaluation metrics were obtained by averaging the model's predictions on test ligands from the last 5 steps (steps 16-20).

6 Active learning experiment on the TYK2 target

For the active learning experiment on the TYK2 target, we used the TYK2 kinase inhibitor dataset released by Google Research[25]. This dataset contains 10,000 ligands with binding free energies calculated using FEP. The dataset was initially constructed from 573 measured ligands found in public patents and was then expanded to 10,000 ligands through R-group assembly and filtering. The binding free energies were calculated using the Relatively Absolute Binding Free Energy (RABFE) protocol[26], which achieved an R^2 of 0.41 with experimental affinities in validation tests. The entire calculation consumed 80,000 GPU hours (equivalent to 9.1 GPU years), which would cost 160,000$.

We implemented an active learning framework to evaluate LigUnity and competing methods. Our goal was to identify ligands with optimal binding free energy within specific FEP calculation constraints. The procedure consisted of the following steps: (1) Sampling a pool of ligands using a defined selection strategy; (2) Calculating binding free energies for selected ligands using the FEP method and incorporating them into the training set (in this study, we directly used existing values from the dataset); (3) Training a model using ligands with known binding free energies; (4) Using the trained model to predict binding affinities for unmeasured ligands; (5) Repeating steps 1-4 until reaching a predetermined number of accessed ligands. This procedure simulates real-world drug discovery scenarios, providing insights into the practical utility of different methods.

For each iteration, we explored 100 ligands, starting with a randomly sampled initial training set of 100 ligands, and we conducted 20 active learning iterations in total. For each iteration, we performed 20 fine-tuning steps on the fine-tuning ligands. The final predictions were obtained by averaging the model's predictions on test ligands from the last 5 steps (steps 16-20). We evaluated each method using two types of metrics: (1) top 1%, 2%, and 5% recall, defined as the ratio of correctly identified top ligands to the total number of top ligands, and (2) r^2, calculated based on the ligands in the test set.

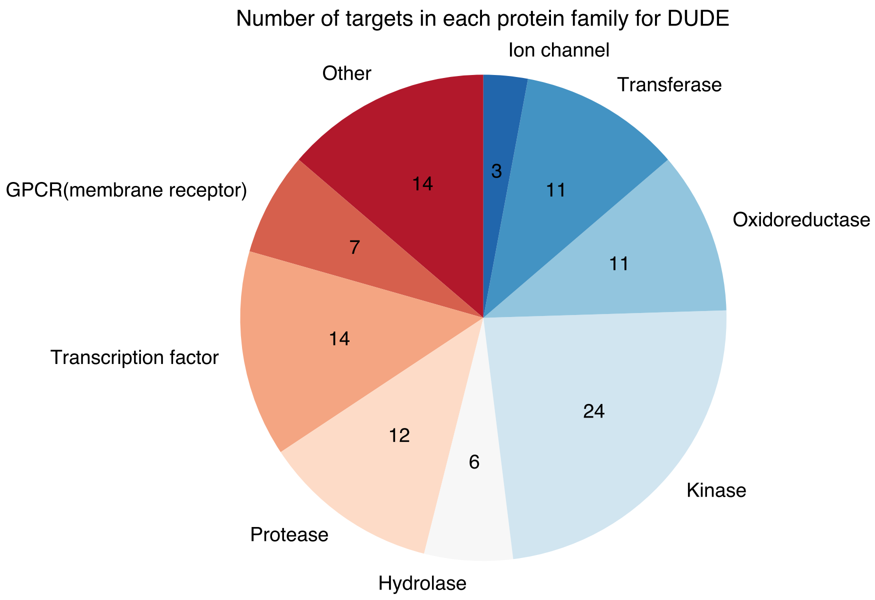

Figure S1: Pie chart showing the number of targets for each protein family in the DUD-E benchmark. The protein families come from ChEMBL[3].

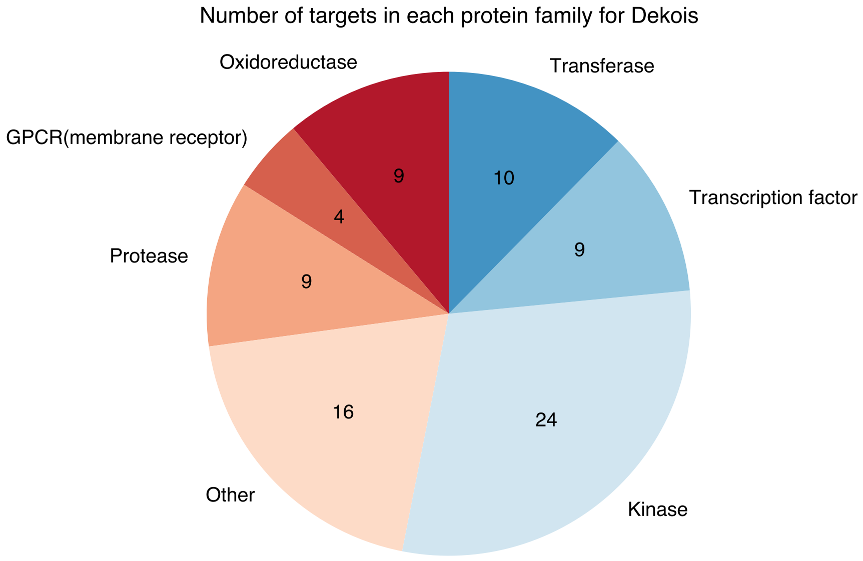

Figure S2: Pie chart showing the number of targets for each protein family in the Dekois 2.0 benchmark. The protein families come from ChEMBL[3].

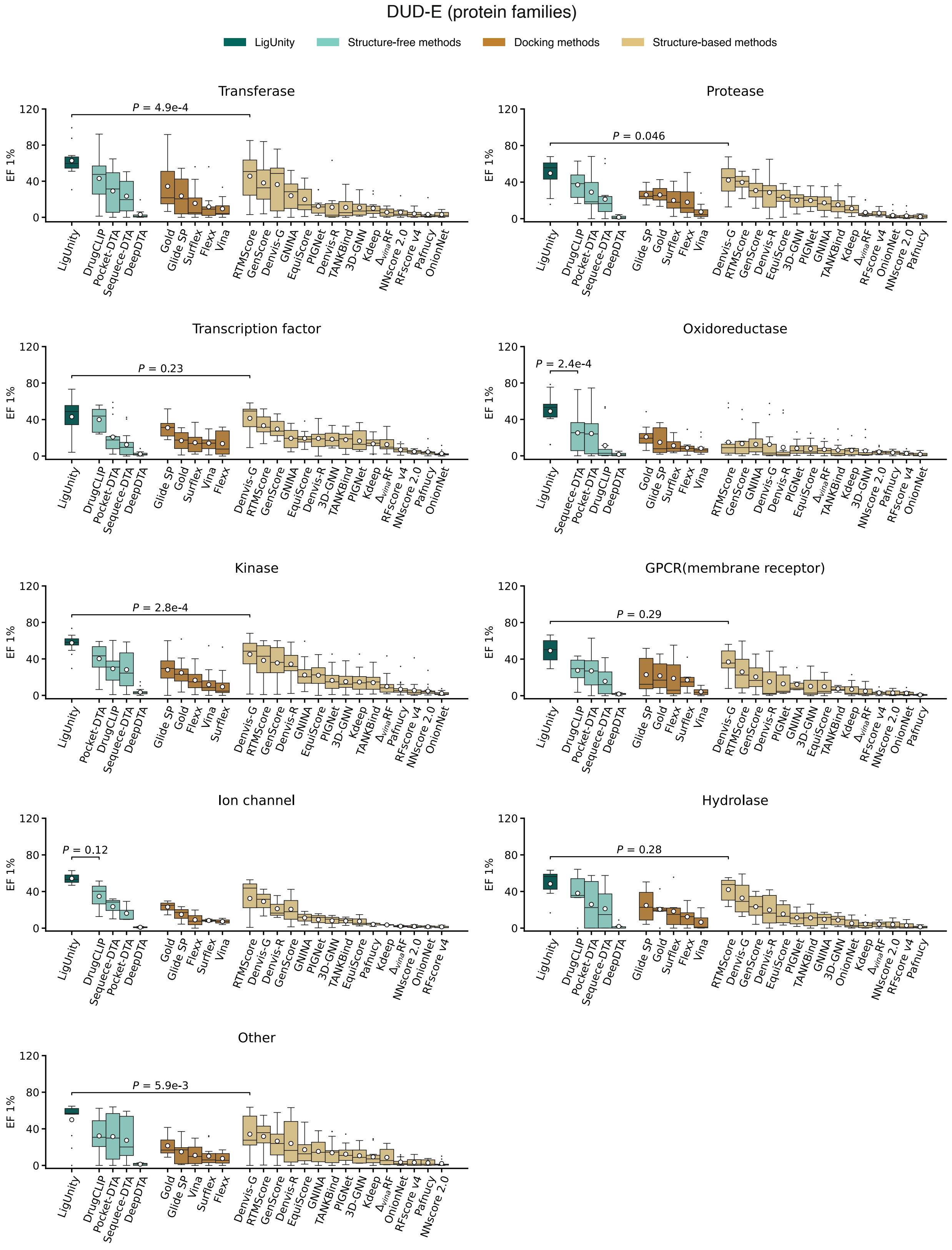

Figure S3: Box plots comparing LigUnity and competing methods on the DUD-E benchmark in terms of enrichment factor (EF) 1%. Targets of different protein families are tested separately.

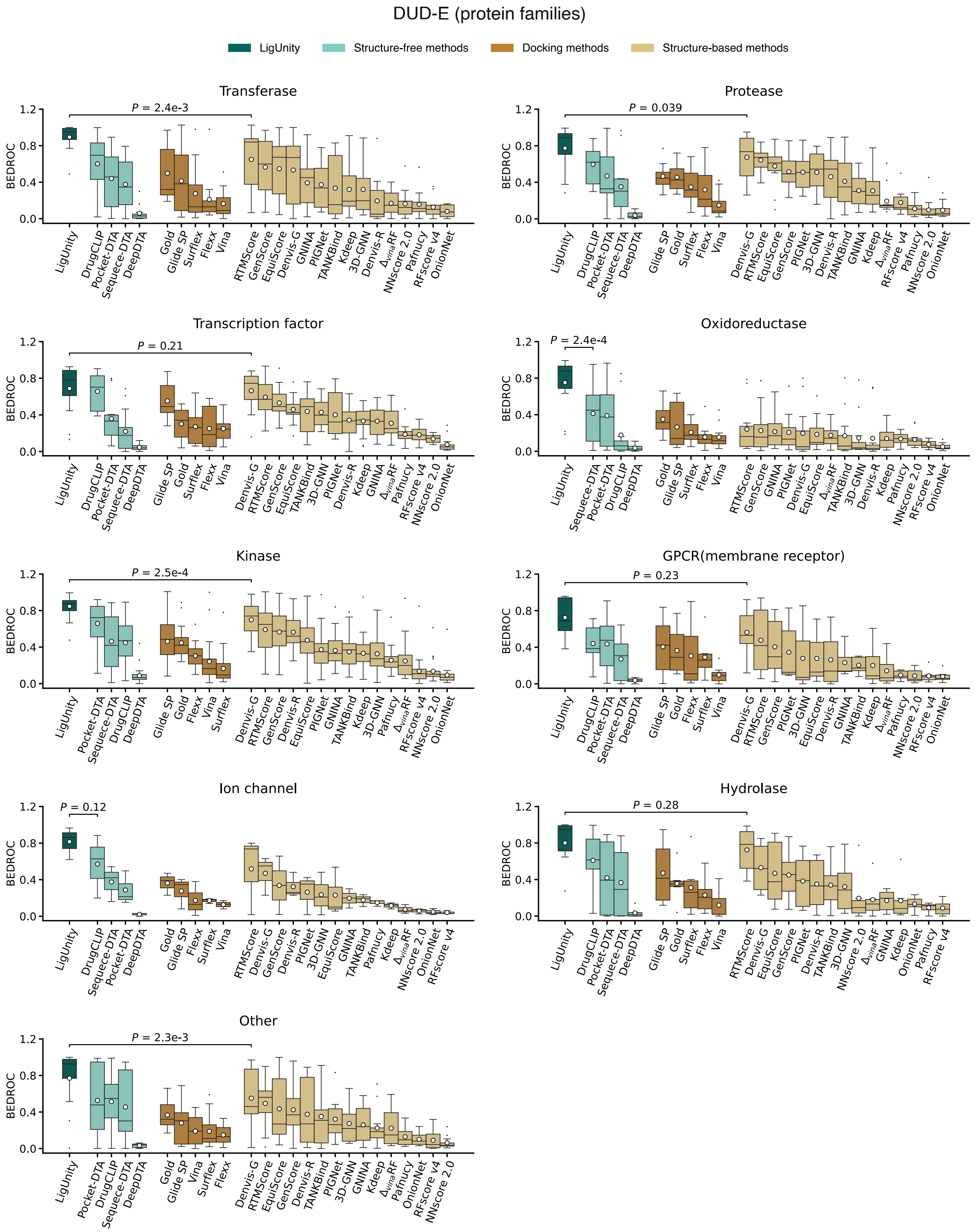

Figure S4: Box plots comparing LigUnity and competing methods on the DUD-E benchmark in terms of BEDROC. Targets of different protein families are tested separately.

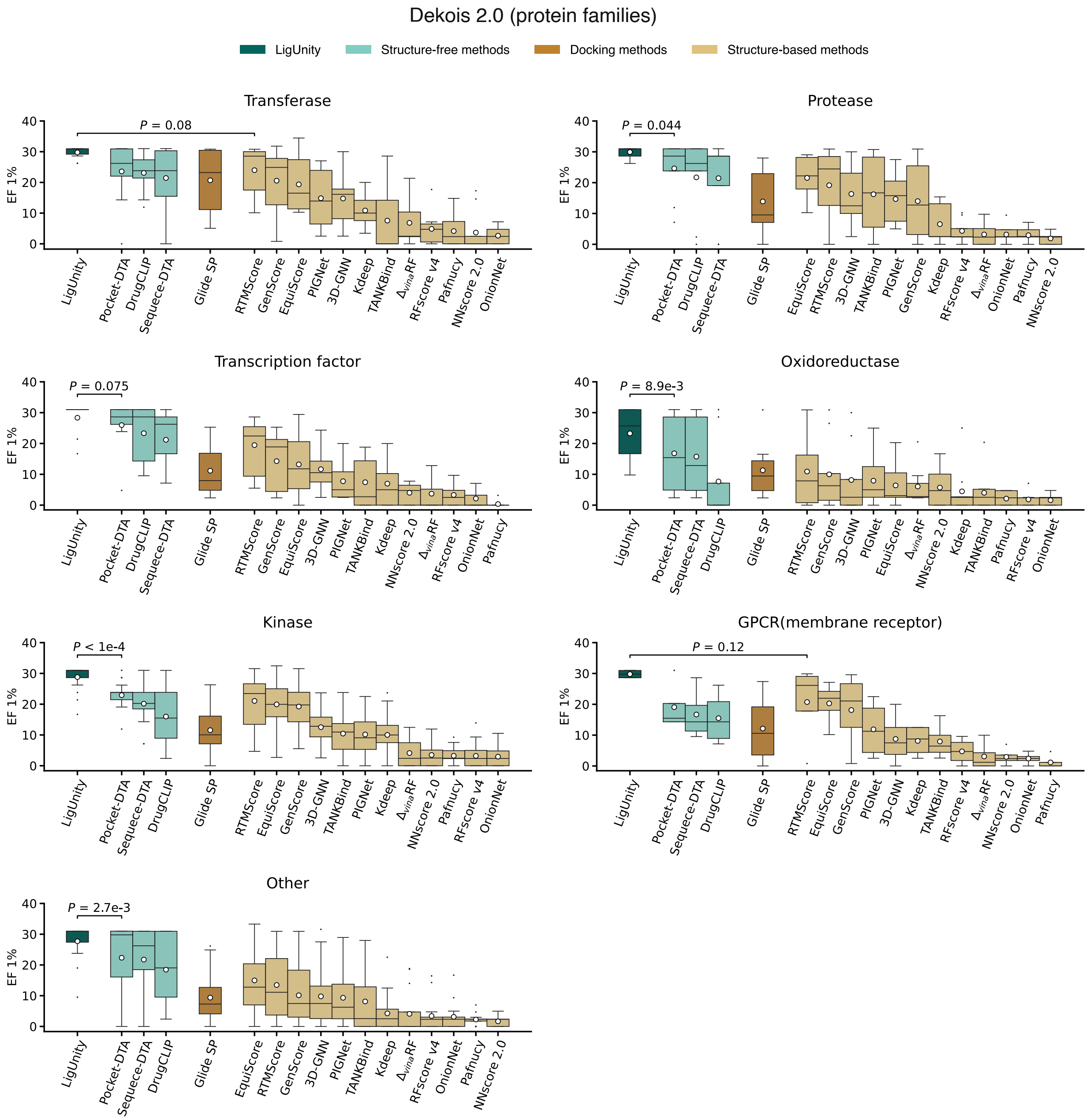

Figure S5: Box plots comparing LigUnity and competing methods on the Dekois 2.0 benchmark in terms of enrichment factor (EF) 1%. Targets of different protein families are tested separately.

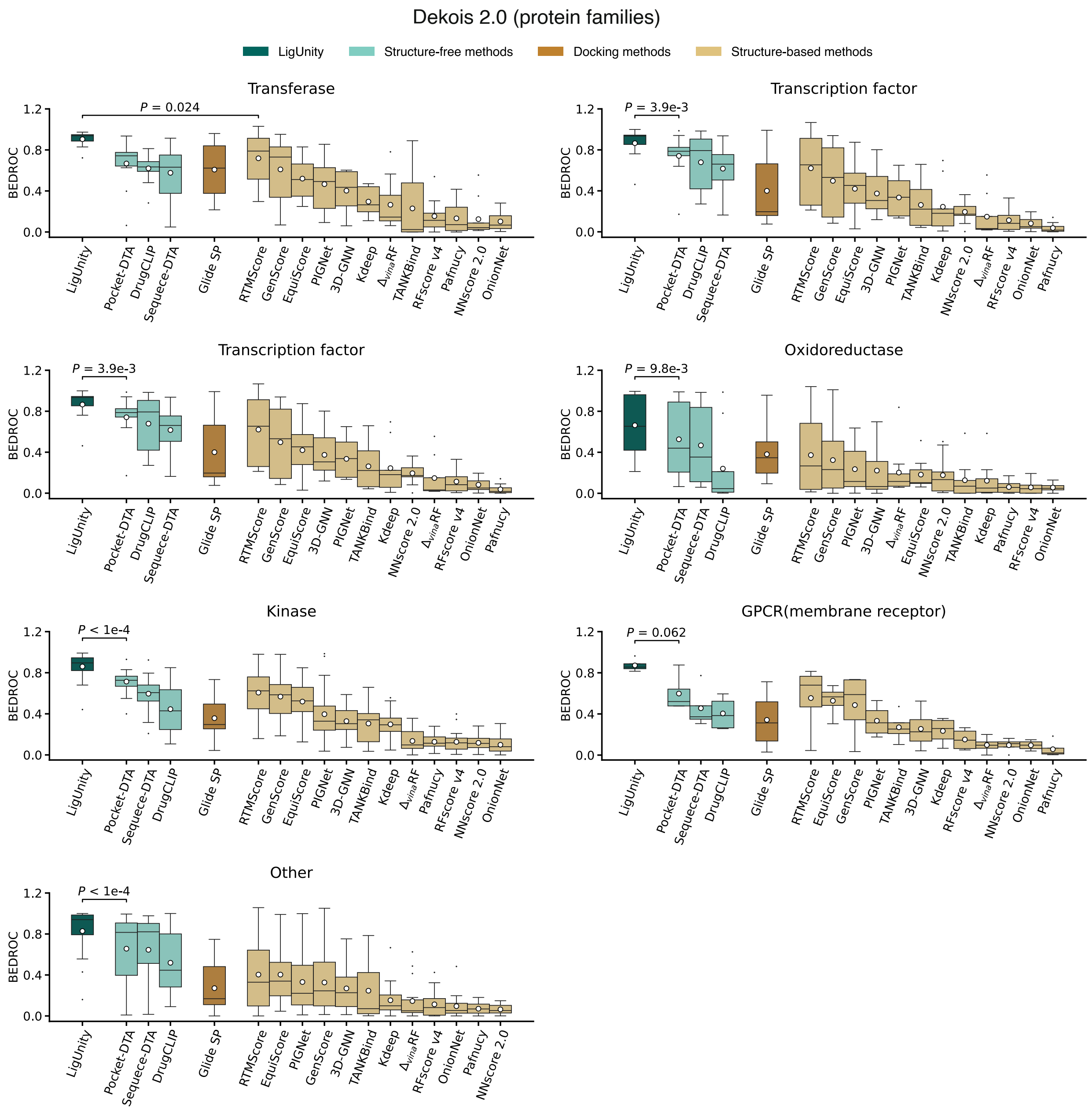

Figure S6: Box plots comparing LigUnity and competing methods on the Dekois 2.0 benchmark in terms of BEDROC. Targets of different protein families are tested separately.

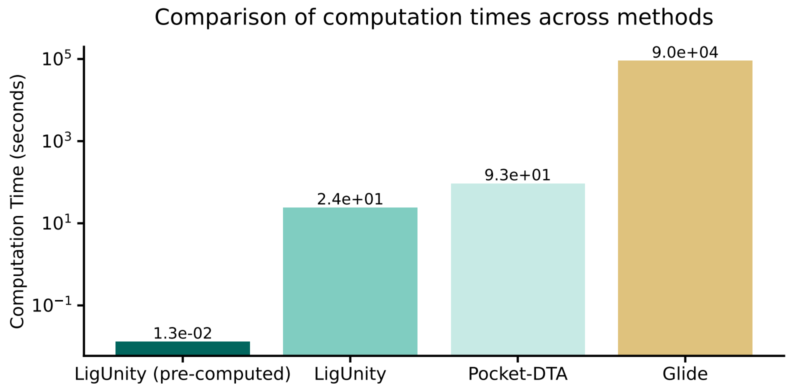

Figure S7: Bar plots compare LigUnity and competing methods based on the computation times for screening 10,000 ligands. Glide-SP's computation time is estimated on a 2.8 GHz Intel Xeon E5-1603 processor, while LigUnity's and Pocket-DTA's times are estimated on a NVIDIA RTX A6000 GPU.

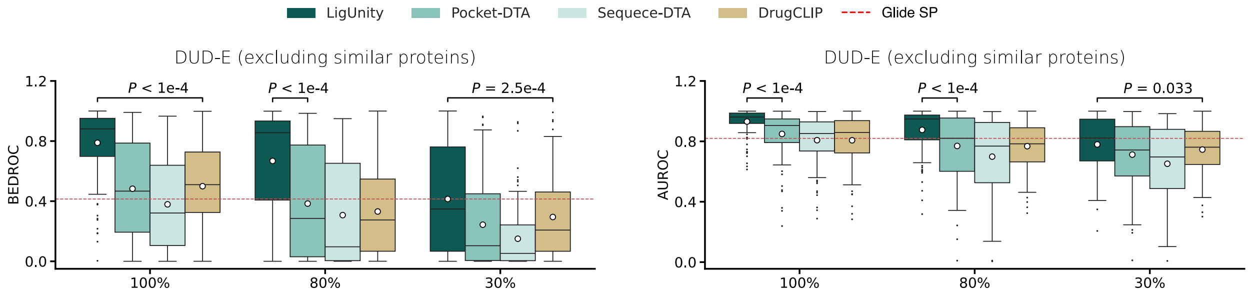

Figure S8: Box plots comparing LigUnity and competing methods on the DUD-E benchmark in terms of BEDROC and AU-ROC using different protein training sets. The *x*-axis denotes the maximum sequence similarity between the training and test sets. The mean values (white dots) are calculated across n = 102 targets.

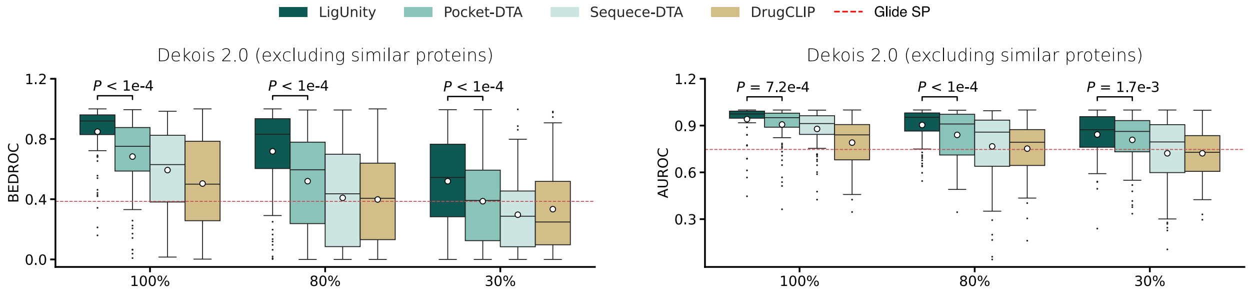

Figure S9: Box plots comparing LigUnity and competing methods on the Dekois 2.0 benchmark in terms of BEDROC and AU-ROC using different protein training sets. The *x*-axis denotes the maximum sequence similarity between the training and test sets. The mean values (white dots) are calculated across n = 81 targets.

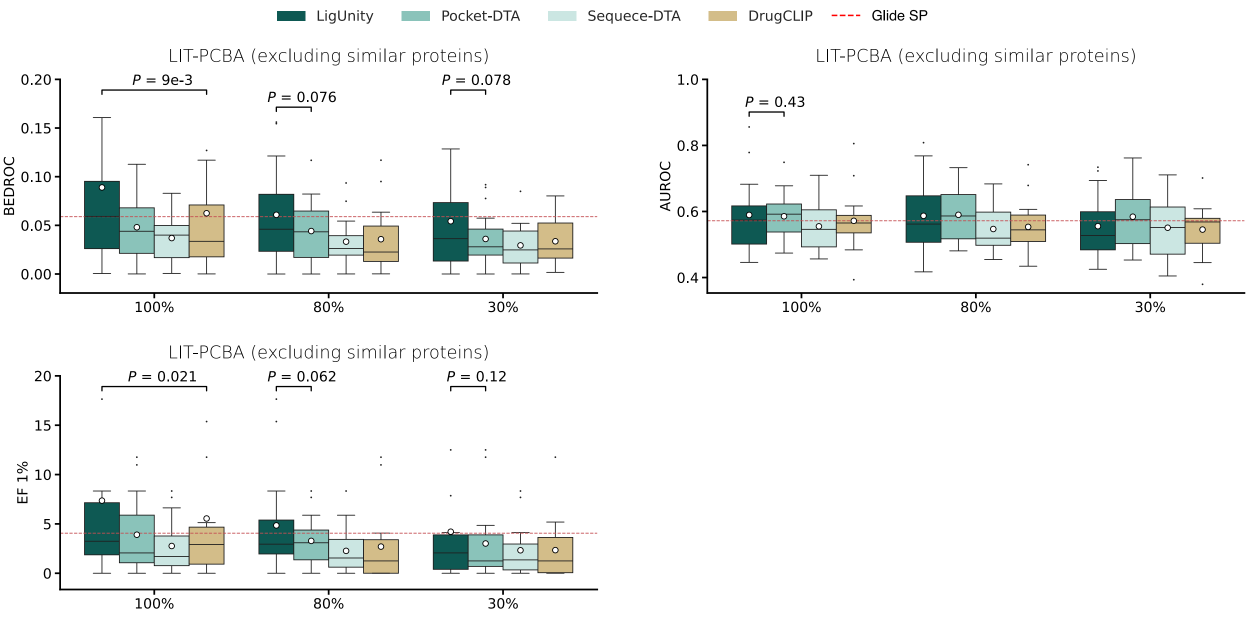

Figure S10: Box plots comparing LigUnity and competing methods on the LIT-PCBA benchmark in terms of enrichment factor (EF) 1%, BEDROC, and AU-ROC using different protein training sets. The *x*-axis denotes the maximum sequence similarity between the training and test sets. The mean values (white dots) are calculated across n = 15 targets.

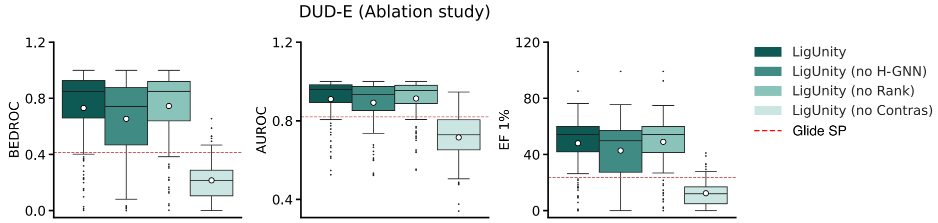

Figure S11: Box plots of ablation study evaluating the contribution of different modules on the DUD-E benchmark in terms of enrichment factor (EF) 1%, BEDROC, and AU-ROC, where "no Contras" means that the contrastive loss for scaffold discrimination are ablated and "no Rank" means that the ranking loss for pharmacophore ranking are ablated. The mean values (white dots) are calculated across n = 102 targets.

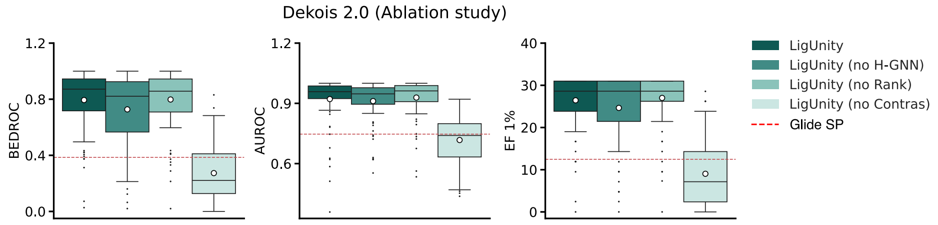

Figure S12: Box plots of ablation study evaluating the contribution of different modules on the Dekois 2.0 benchmark in terms of enrichment factor (EF) 1%, BEDROC, and AU-ROC, where "no Contras" means that the contrastive loss for scaffold discrimination are ablated and "no Rank" means that the ranking loss for pharmacophore ranking are ablated. The mean values (white dots) are calculated across n = 81 targets.

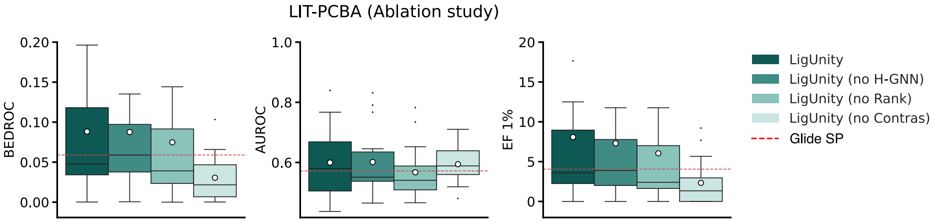

Figure S13: Box plots of ablation study evaluating the contribution of different modules on the LIT-PCBA benchmark in terms of enrichment factor (EF) 1%, BEDROC, and AU-ROC, where "no Contras" means that the contrastive loss for scaffold discrimination are ablated and "no Rank" means that the ranking loss for pharmacophore ranking are ablated. The mean values (white dots) are calculated across n = 15 targets.

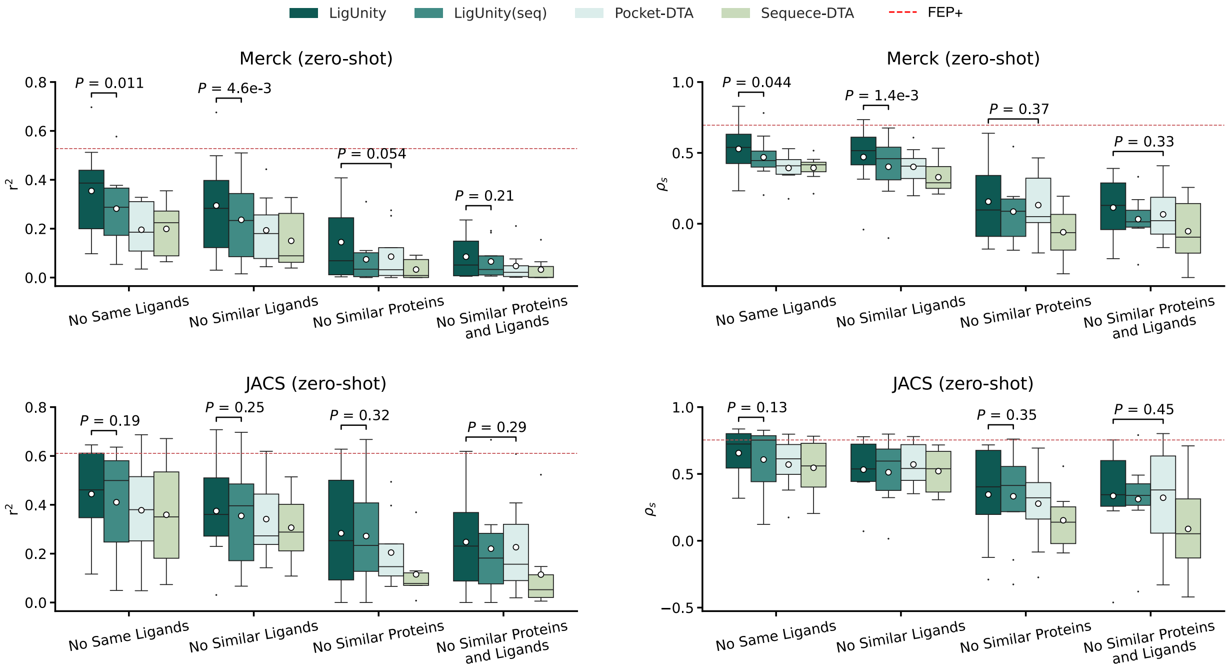

Figure S14: Box plots comparing LigUnity and competing methods across different settings on Merck and JACS benchmarks in terms of r^2 and Spearman's rank correlation (ρ_s). The mean values (white dots) are calculated across n = 8 targets.

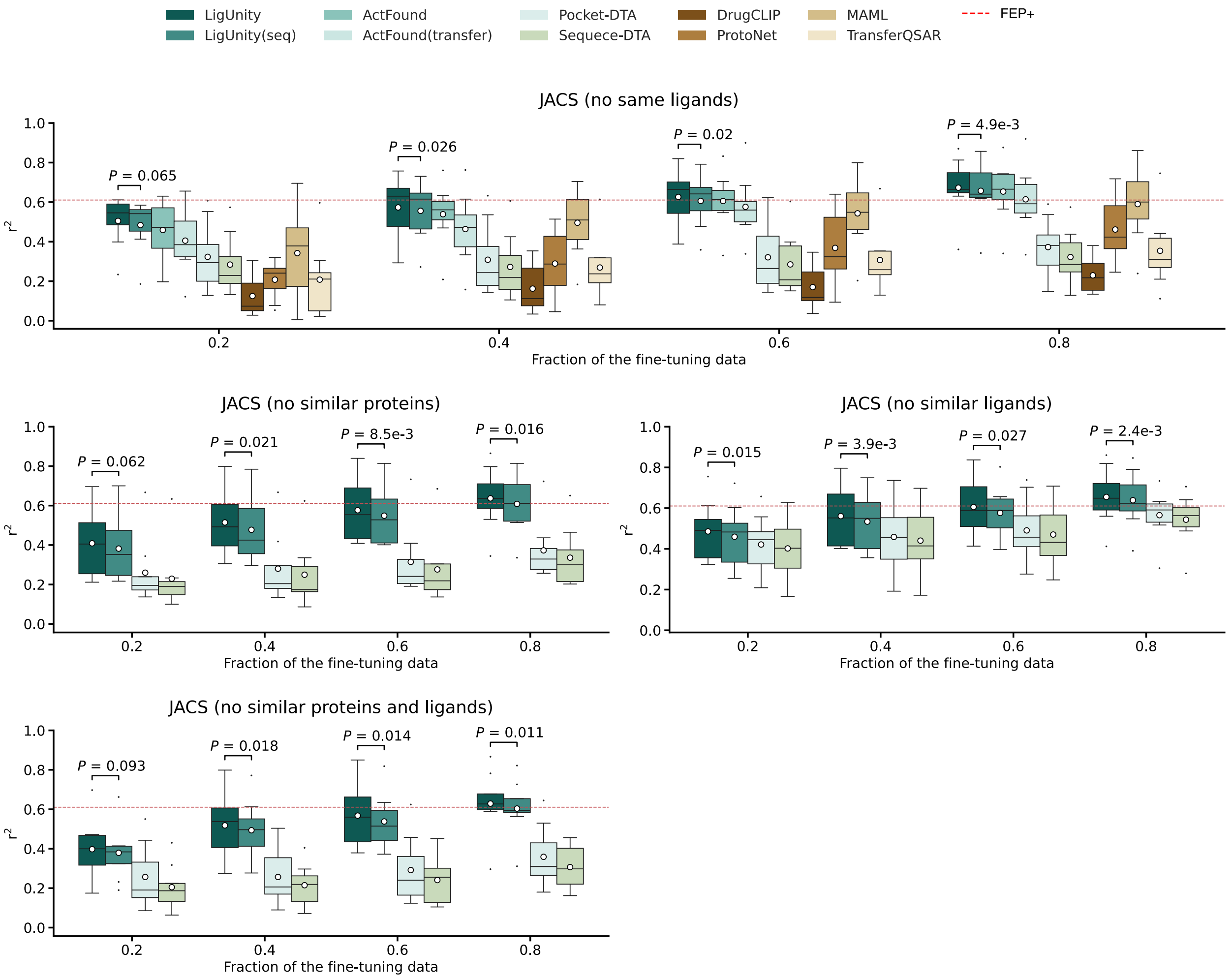

Figure S15: Box plots comparing LigUnity and competing methods across different settings on the JACS benchmark in terms of r^2 and Spearman's rank correlation (ρ_s) when 20%, 40%, 60%, 80% of the experimental binding affinities are used for fine-tuning. The mean values (white dots) are calculated across n = 8 targets.

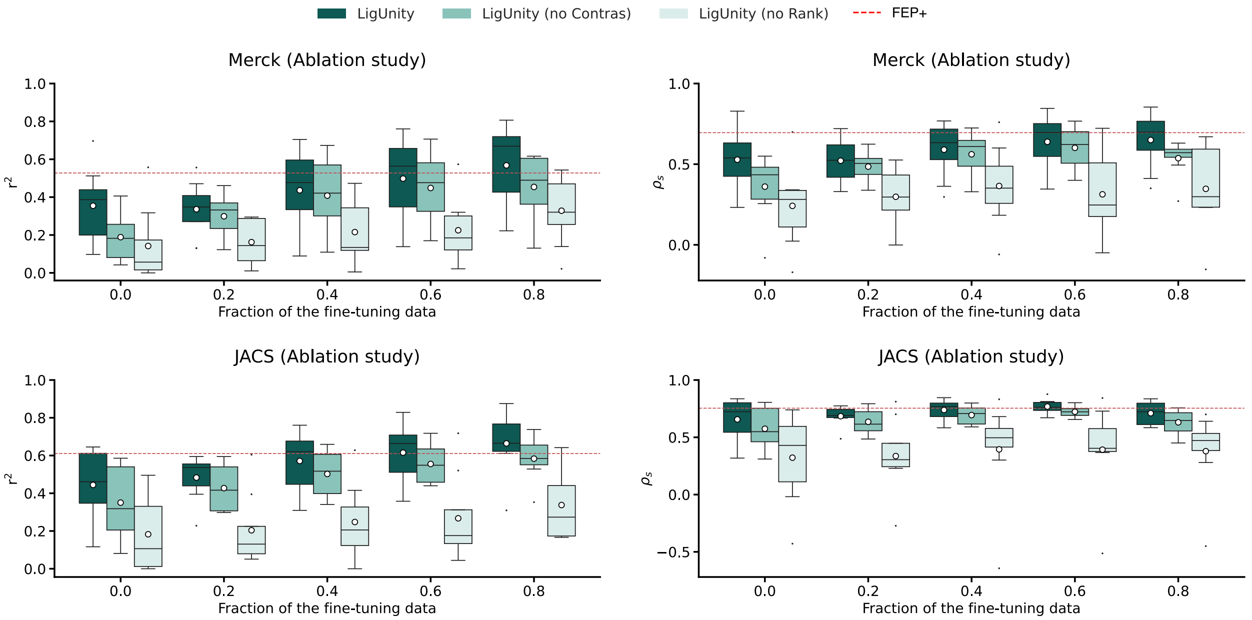

Figure S16: Box plots of ablation study evaluating the contribution of different modules on Merck and JACS benchmarks in terms of r^2 and Spearman's rank correlation (ρ_s) when 0% (zero-shot), 20%, 40%, 60%, 80% of the experimental binding affinities are used for fine-tuning, where "no Contras" means that the contrastive loss for scaffold discrimination are ablated and "no Rank" means that the ranking loss for pharmacophore ranking are ablated. The mean values (white dots) are calculated across n = 8 targets.

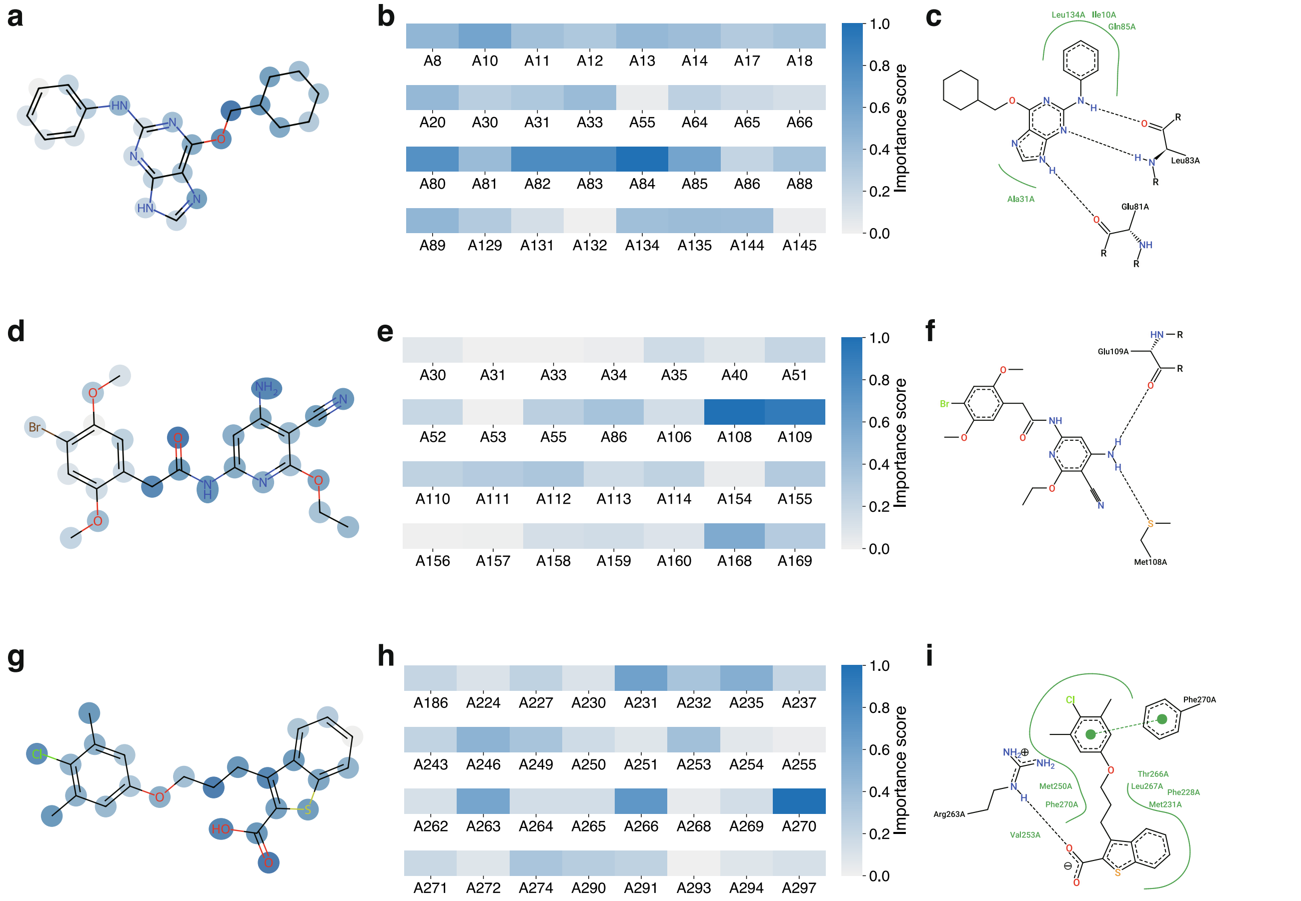

Figure S17: Case study on the CDK2 target **(a,b,c)**, JNK1 target **(d,e,f)**, MCL1 **(g, h, i)** target showing the importance score of each ligand atom **(a,d,g)** and pocket residue **(b,e,h)** predicted by LigUnity, and 2D interaction graph **(c,f,i)** showing the non-covalent interaction between the ligand and pocket.

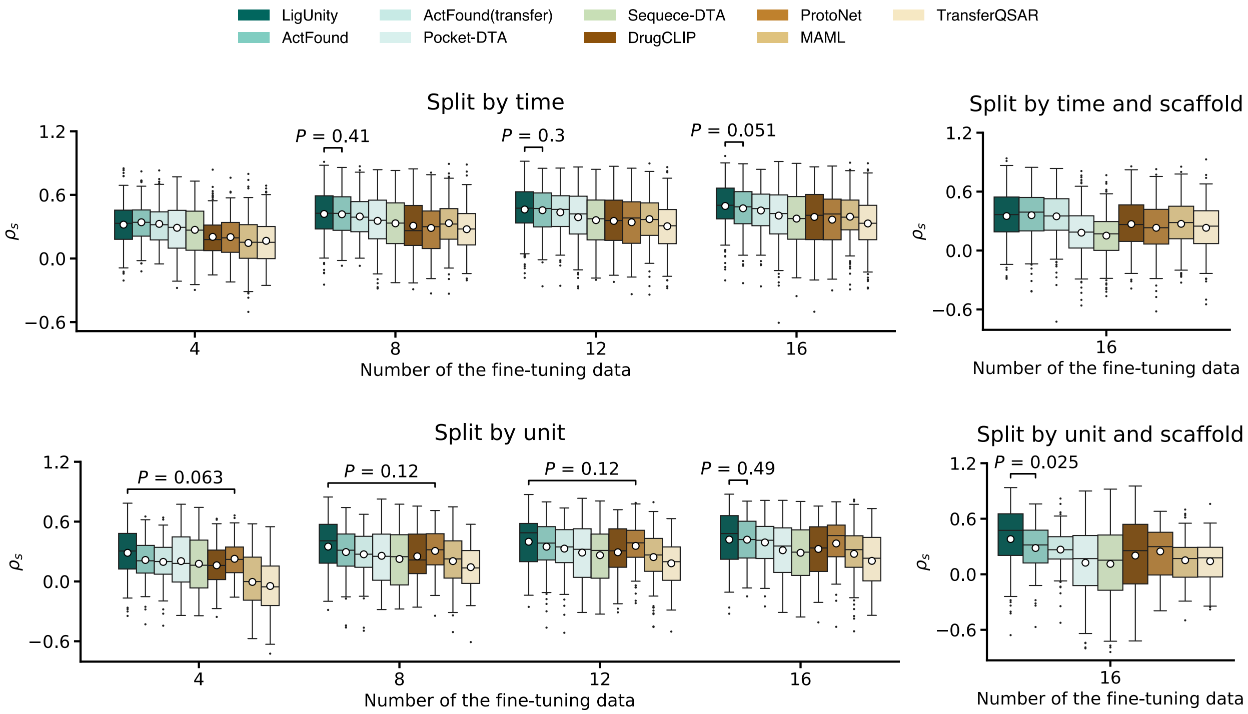

Figure S18: Box plots comparing the binding affinity prediction on the split-by-time and split-by-unit setting in terms of ρ_s when 4, 8, 12, and 16 of the experimental binding affinity is used for fine-tuning. The mean values (white dots) are calculated across n = 161 assays for split-by-time setting and n = 65 assays for split-by-time setting.

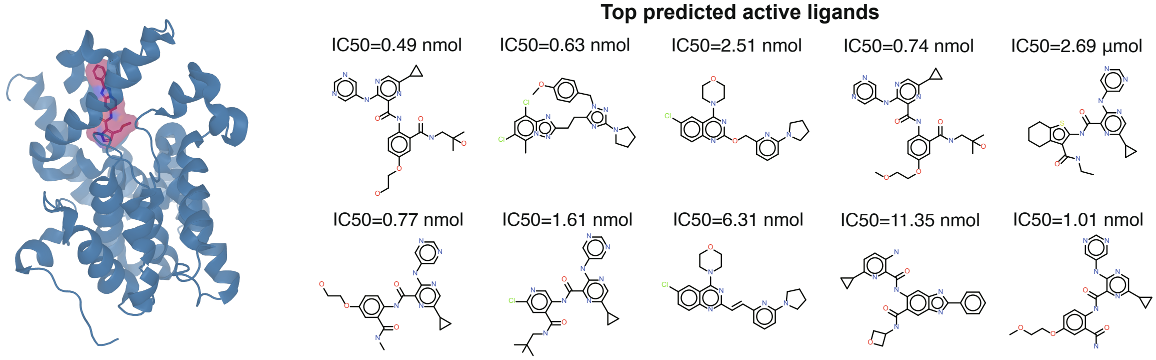

Figure S19: 3D structure for the PDE10A target and its active ligand, and the top 10 ligands ranked by affinities predicted using LigUnity, along with their experimental IC50 values.

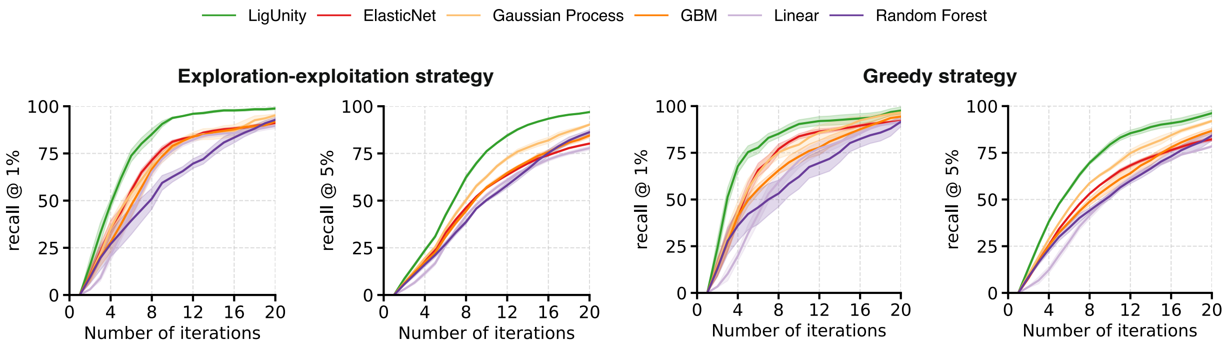

Figure S20: Plots comparing LigUnity and competing methods on the TYK2 dataset in terms of top 1% recall and top 5% recall when using the exploration-exploitation strategy (left) and greedy selection strategy (right).

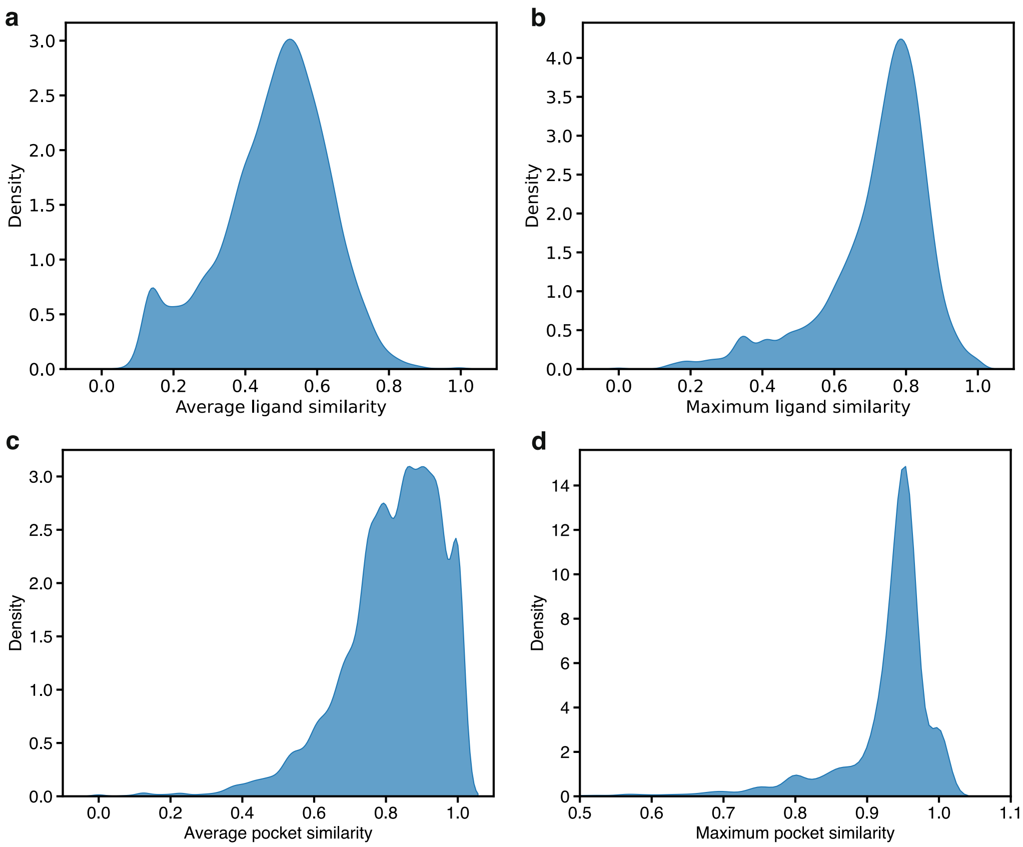

Figure S21: **(a,)** Kernel density estimate (KDE) plot showing average pairwise similarity between any two ligands in an assay. **(b,)** KDE plot showing average similarity between each ligand and its most similar counterpart (nearest neighbor) in an assay. **(c,)** KDE plot showing average pairwise similarity between any two pockets in an assay. **(d,)** KDE plot showing average similarity between each pocket and its most similar counterpart (nearest neighbor) in an assay. The ligand similarity is computed using tanimoto similarity (ECFP4) and the pocket similarity is computed using sequence similarity.

Table S1: Performance comparison between LigUnity and BIND[27] on virtual screening benchmarks (DUD-E, LIT-PCBA, and DEKOIS 2.0) in terms of EF 1%, AUROC, and BEDROC score ($\alpha = 80.5$), evaluated under sequence similarity exclusion (90% similarity to test data).

Method	Metric	DUD-E	LIT-PCBA	DEKOIS 2.0
BIND	EF1%	26.390 (2.008)	4.450 (1.170)	15.190 (1.060)
	AUROC	0.826 (0.018)	0.564 (0.030)	0.832 (0.018)
	BEDROC	0.430 (0.030)	0.047 (0.010)	0.453 (0.029)
LigUnity	EF1%	44.781 (2.243)	4.685 (1.402)	23.944 (0.992)
	AUROC	0.871 (0.016)	0.554 (0.015)	0.882 (0.014)
	BEDROC	0.670 (0.032)	0.060 (0.015)	0.698 (0.029)

Table S2: Performance comparison between LigUnity and PBCNet[28] on hit-to-lead optimization tasks in terms of Pearson correlation using Merck and JACS benchmarks. Results are shown for zero-shot (0), 6-shot, and 10-shot settings, reporting Pearson's correlation coefficients. LigUnity is trained following PBCNet's setting which excludes training assays (congeneric series in PBCNet) identical to assays in FEP benchmarks. We consider two assays identical if they share the same protein and ligands.

	Merck			JACS		
Method	0	6	10	0	6	10
PBCNet	0.468	0.592	0.646	0.648	0.729	0.731
LigUnity	0.494	0.631	0.676	0.690	0.746	0.775

Table S3: Validation of LigUnity's ability to learn protein-ligand interaction patterns through targeted residue masking experiments. Performance metrics (square of Pearson correlation r^2 and Spearman correlation ρ with standard deviations) are shown for Merck and JACS benchmarks under three conditions: (1) masking key interaction residues identified via ProteinPlus[29], (2) masking randomly selected residues, and (3) no masking.

	Merck		JACS	
Method	r^2	ρ_s	r^2	ρ_s
Mask key residue	0.323 (0.070)	0.484 (0.066)	0.354 (0.073)	0.522 (0.088)
Mask random residue	0.328 (0.067)	0.497 (0.062)	0.391 (0.063)	0.575 (0.074)
No mask	0.333 (0.068)	0.499 (0.063)	0.389 (0.064)	0.578 (0.074)

Table S4: Performance comparison of LigUnity under different data curation strategies on virtual screening benchmarks in terms of EF 1%, AUROC, and BEDROC score ($\alpha = 80.5$). "LigUnity (strict ligand)" removes assays with multiple pockets (> 10 Å apart), and "LigUnity (strict pocket)" applies a 100% Tanimoto similarity threshold for pocket assignment.

**Benchmark**	**Metric**	**LigUnity**	**LigUnity (strict ligand)**	**LigUnity (strict pocket)**
DUDE	BEDROC	0.789 (0.023)	0.725 (0.026)	0.732 (0.028)
	AUROC	0.931 (0.009)	0.903 (0.013)	0.905 (0.011)
	EF1	52.042 (1.688)	47.590 (1.865)	48.374 (2.013)
LIT-PCBA	BEDROC	0.089 (0.027)	0.082 (0.026)	0.082 (0.021)
	AUROC	0.589 (0.029)	0.553 (0.029)	0.570 (0.025)
	EF1	7.359 (2.488)	6.803 (2.626)	7.320 (2.276)
DEKOIS 2.0	BEDROC	0.849 (0.021)	0.776 (0.026)	0.778 (0.025)
	AUROC	0.941 (0.011)	0.916 (0.012)	0.910 (0.011)
	EF1	28.212 (0.546)	25.995 (0.863)	26.205 (0.785)

References

1. Lin, Z. *et al.* Evolutionary-scale prediction of atomic-level protein structure with a language model. *Science* **379**, 1123–1130 (2023).
2. Gilson, M. K. *et al.* Bindingdb in 2015: a public database for medicinal chemistry, computational chemistry and systems pharmacology. *Nucleic acids research* **44**, D1045–D1053 (2016).

3. Zdrazil, B. *et al.* The ChEMBL Database in 2023: a drug discovery platform spanning multiple bioactivity data types and time periods. *Nucleic acids research* **52**, D1180–D1192 (2024).

4. Feng, B. *et al.* A bioactivity foundation model using pairwise meta-learning. *Nat. Mach. Intell.* DOI: 10.1038/s42256-024-00876-w (2024).

5. Zardecki, C., Dutta, S., Goodsell, D. S., Voigt, M. & Burley, S. K. Rcsb protein data bank: A resource for chemical, biochemical, and structural explorations of large and small biomolecules. *J. Chem. Educ.* **93**, 569–575, DOI: 10.1021/acs.jchemed.5b00404 (2016).

6. Abramson, J. *et al.* Accurate structure prediction of biomolecular interactions with alphafold 3. *Nature* **630**, 493 (2024).

7. Maggiora, G., Vogt, M., Stumpfe, D. & Bajorath, J. Molecular similarity in medicinal chemistry. *J. Medicinal Chem.* **57**, 3186–3204, DOI: 10.1021/jm401411z (2014). PMID: 24151987, https://doi.org/10.1021/jm401411z.

8. Yang, J., Roy, A. & Zhang, Y. Biolip: a semi-manually curated database for biologically relevant ligand–protein interactions. *Nucleic acids research* **41**, D1096–D1103 (2012).

9. Martin, E. J. *et al.* All-assay-Max2 pQSAR: activity predictions as accurate as four-concentration ic50s for 8558 novartis assays. *J. chemical information modeling* **59**, 4450–4459 (2019).

10. Berman, H. M. *et al.* The Protein Data Bank. *Nucleic acids research* **28**, 235–242 (2000).

11. Shen, C. *et al.* Boosting protein–ligand binding pose prediction and virtual screening based on residue–atom distance likelihood potential and graph transformer. *J. Medicinal Chem.* **65**, 10691–10706 (2022).

12. Gao, B. *et al.* DrugCLIP: Contrastive protein-molecule representation learning for virtual screening. *Adv. Neural Inf. Process. Syst.* (2023).

13. Wang, R., Fang, X., Lu, Y. & Wang, S. The pdbbind database: Collection of binding affinities for protein-ligand complexes with known three-dimensional structures. *J. Medicinal Chem.* **47**, 2977–2980 (2004).

14. Mysinger, M. M., Carchia, M., Irwin, J. J. & Shoichet, B. K. Directory of useful decoys, enhanced (dud-e): Better ligands and decoys for better benchmarking. *J. Medicinal Chem.* **55**, 6582–6594 (2012).

15. Vogel, S. M., Bauer, M. R. & Boeckler, F. M. Dekois: Demanding evaluation kits for objective in silico screening–a versatile tool for benchmarking docking programs and scoring functions. *J. chemical information modeling* **51**, 2650–2665 (2011).

16. Tran-Nguyen, V.-K., Jacquemard, C. & Rognan, D. Lit-pcba: an unbiased data set for machine learning and virtual screening. *J. chemical information modeling* **60**, 4263–4273 (2020).

17. Krasoulis, A., Antonopoulos, N., Pitsikalis, V. & Theodorakis, S. Denvis: scalable and high-throughput virtual screening using graph neural networks with atomic and surface protein pocket features. *J. Chem. Inf. Model.* **62**, 4642–4659 (2022).

18. Cao, D. *et al.* Generic protein–ligand interaction scoring by integrating physical prior knowledge and data augmentation modelling. *Nat. Mach. Intell.* 1–13 (2024).

19. Shen, C. *et al.* A generalized protein–ligand scoring framework with balanced scoring, docking, ranking and screening powers. *Chem. Sci.* **14**, 8129–8146 (2023).

20. Wang, L. *et al.* Accurate and reliable prediction of relative ligand binding potency in prospective drug discovery by way of a modern free-energy calculation protocol and force field. *J. Am. Chem. Soc.* **137**, 2695–2703 (2015).

21. Schindler, C. E. *et al.* Large-scale assessment of binding free energy calculations in active drug discovery projects. *J. Chem. Inf. Model.* **60**, 5457–5474 (2020).

22. Lu, C. *et al.* OPLS4: Improving force field accuracy on challenging regimes of chemical space. *J. chemical theory computation* **17**, 4291–4300 (2021).

23. Ross, G. A. *et al.* The maximal and current accuracy of rigorous protein-ligand binding free energy calculations. *Commun. Chem.* **6**, 222 (2023).

24. Tosstorff, A. *et al.* A high quality, industrial data set for binding affinity prediction: performance comparison in different early drug discovery scenarios. *J. Comput. Mol. Des.* **36**, 753–765 (2022).

25. Thompson, J. *et al.* Optimizing active learning for free energy calculations. *Artif. Intell. Life Sci.* **2**, 100050 (2022).

26. Rocklin, G. J., Mobley, D. L. & Dill, K. A. Separated topologies—a method for relative binding free energy calculations using orientational restraints. *The J. chemical physics* **138** (2013).

27. Lam, H. Y. I., Guan, J. S., Ong, X. E., Pincket, R. & Mu, Y. Protein language models are performant in structure-free virtual screening. *Briefings Bioinforma.* **25**, bbae480 (2024).

28. Yu, J. *et al.* Computing the relative binding affinity of ligands based on a pairwise binding comparison network. *Nat. Comput. Sci.* **3**, 860–872 (2023).

29. Schöning-Stierand, K. *et al.* Proteins plus: interactive analysis of protein–ligand binding interfaces. *Nucleic acids research* **48**, W48–W53 (2020).
